# *Drosophila suzukii* and *Drosophila melanogaster* prefer distinct microbial and plant aroma compounds in a complex fermented matrix

**DOI:** 10.1016/j.isci.2024.111141

**Published:** 2024-10-10

**Authors:** Maria C. Dzialo, Somasundar Arumugam, Supinya Piampongsant, Lloyd Cool, Christophe Vanderaa, Beatriz Herrera-Malaver, Tomas Opsomer, Wim Dehaen, Tom Wenseleers, Miguel Roncoroni, Amani Alawamleh, Felix Wäckers, Bart Lievens, Bill S. Hansson, Karin Voordeckers, Silke Sachse, Kevin J. Verstrepen

**Affiliations:** 1 VIB – KU Leuven Center for Microbiology, Gaston Geenslaan 1, 3001 Leuven, Belgium; 2 CMPG Laboratory of Genetics and Genomics, Department M2S, KU Leuven, Gaston Geenslaan 1, 3001 Leuven, Belgium; 3 Research Group Olfactory Coding, Max Planck Institute for Chemical Ecology, Hans-Knoell-Str, 8, 07745 Jena, Germany; 4 Max Planck Center Next Generation Insect Chemical Ecology (nGICE), Hans-Knoell-Str, 8, 07745 Jena, Germany; 5 Laboratory of Socioecology and Social Evolution, Department of Biology, KU Leuven, Naamsestraat 59, 3000 Leuven, Belgium; 6 Sustainable Chemistry for Metals and Molecules, Department of Chemistry, KU Leuven, Celestijnenlaan 200F, 3001 Leuven, Belgium; 7 Biobest NV, Ilse Velden 18, 2260 Westerlo, Belgium; 8 University of Molise, Department of Agricultural, Environmental and Food Sciences, Via De Sanctis 1, 86100 Campobasso, Italy; 9 Lancaster Environment Centre, Lancaster University, Lancaster LA1 4YQ, UK; 10 CMPG Laboratory for Process Microbial Ecology and Bioinspirational Management (PME&BIM), Department M2S, KU Leuven, Willem De Croylaan 46, 3001 Leuven, Belgium; 11 Department of Evolutionary Neuroethology, Max Planck Institute for Chemical Ecology, Hans-Knoell-Str, 8, 07745 Jena, Germany

**Keywords:** Ethology, Sensory neuroscience, Microbiology

## Abstract

Volatile aroma compounds are important chemical cues for insects. Behavioral responses to specific odors differ strongly between insect species, and the exact causative molecules are often unknown. Beer is frequently used in insect traps because it combines hundreds of plant and microbial aromas that attract many insects. Here, we analyzed responses of the pest fruit fly *Drosophila suzukii* and benign *Drosophila melanogaster* to beers with different chemical compositions. Using extensive chemical and behavioral assays, we identified ecologically relevant chemicals that influence drosophilid behavior and that induce different odor-evoked activity patterns in the antennal lobe of the two species obtained by functional imaging. Specific mixes of compounds increased the species-specificity and sex-specificity of lures in both laboratory and greenhouse settings. Together, our study shows how examining insect responses to highly complex natural mixtures of aroma compounds provides insight into insect-specific behavioral responses and also opens avenues for improved pest control.

## Highlights

• *D. suzukii* and *D. melanogaster* prefer beers with different chemical compositions
• Specific beer compounds lead to distinct attraction and repulsion in both species
• Compounds cause species-specific odor-evoked neuronal responses in antennal lobe
• Beer compounds can be used to develop species-specific synthetic lures

## Graphical abstract

**Figure:**
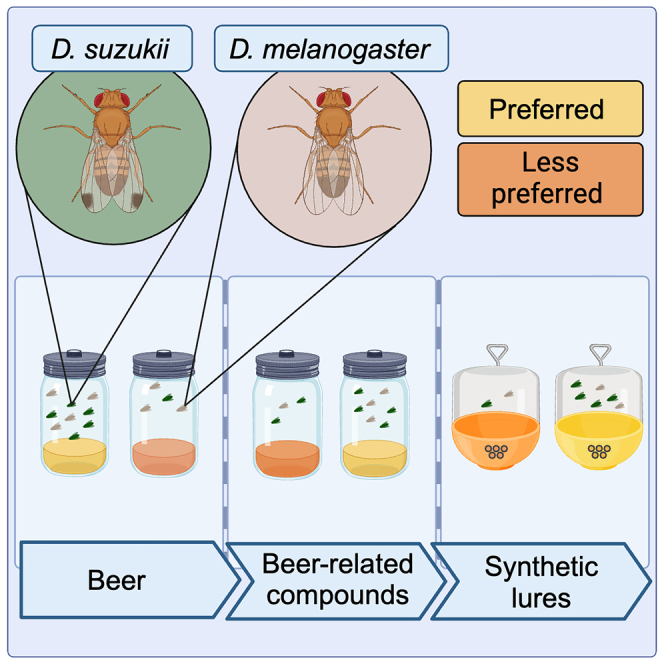

## Introduction

Volatile-mediated interactions among plants, microbes, and insects represent an important facet in ecology.^1^ Odor-based interactions between different living organisms are important to understand complex ecological interactions and also serve as a model for neurobiological studies, where the molecular toolbox of model organisms like *Drosophila melanogaster* is used to unravel the links between volatile cues, neuronal responses, and behavior. Moreover, insights into odor-driven insect behavior are exploited in agriculture by developing traps that help with monitoring and control of pest insects.^2^ The majority of studies and applications of volatile signals in agriculture to date focus on signal molecules produced by plants and insects.^3^^,^^4^ Interestingly, recent studies have shown that microbes, including yeasts and bacteria, may also directly emit or alter the emission and composition of volatile signals that are used as insect semiochemicals.^1^^,^^5^^,^^6^^,^^7^ However, the enormous range of volatiles from plants and microbes that insects encounter in their natural habitats remains largely underexplored. Similarly, our understanding of the differences in the response of different insect species to the same volatiles is also limited.

*Drosophila suzukii*, commonly known as the spotted-wing drosophila, is an important fruit crop pest that prefers to lay eggs in, and cause severe damage to, soft-skinned fruits like cherries, blackberries, blueberries, raspberries, and grapes.^8^*D. suzukii* is native to Asia but has become established as an extremely severe pest throughout the northern hemisphere over the last decade.^8^ Unlike most other drosophilids that are attracted to rotting fruit that has no commercial value,^9^^,^^10^ *D. suzukii* primarily lays eggs in fresh, ripening, or ripe fruit.^8^^,^^11^ This preference for ripe(ning) fruit as oviposition target has been linked to changes in olfaction, taste, and mechanosensory responses in *D. suzukii* compared to *D. melanogaster*.^12^^,^^13^^,^^14^^,^^15^^,^^16^ Additionally, in contrast to *D. melanogaster*, female *D. suzukii* have large, serrated ovipositors, enabling penetration of intact fruit skins and subsequent egg deposition.^17^ Once eggs hatch, larval and pupal development of the flies renders the fruit unmarketable. The physical damage to the fruit also increases its susceptibility to microbial infection. The ability of *D. suzukii* to infest a broad range of fruit crops, together with its high reproduction rate and short life cycle, makes this pest a serious threat to fruit farming wherever the species is encountered. Worldwide invasion of this fly has resulted in millions in revenue loss over the last few years.^18^ Moreover, global warming appears to increase *D. suzukii*’s invasive range and results in its appearance earlier in the season, also impacting native insects.^19^

Detection of key ecologically relevant odorants drives adaptative behaviors in *Drosophila* such as the search for a mating partner,^20^ oviposition site selection,^13^^,^^21^ and others. Despite the ecological importance of responding appropriately to the complex chemical environment insects are confronted with, our understanding of the precise chemical cues that drive *D. suzukii* behavior remains limited. As a result of not knowing these chemical cues, commercially available monitoring traps for *D. suzukii* contain attractants that lack selectivity and hence trap significant numbers of non-target insects, particularly other drosophilids.^22^^,^^23^^,^^24^ Sorting through this so-called by-catch is time-intensive and costly. Importantly, it also complicates identification of *D. suzukii* without magnification, particularly for fruit growers lacking entomology expertise. Moreover, despite all efforts, the efficacy of the currently available insect lures is often frustratingly low, in particular because of low attractant specificity.^18^ Hence, the discovery and identification of *D. suzukii*-specific attractants is not only interesting from an ecological and neurobiological perspective, but may also open avenues to develop more effective and specific monitoring traps.

Many studies that investigate odor-driven insect behavior focus on only one or a few monomolecular compounds, whereas in natural settings, volatile-mediated interactions are generally much more complex, with a multitude of volatile aroma compounds involved, some of which at extremely low concentrations.^25^ Both the host fruit as well as microbial fermentation products have been shown to play a crucial role in attracting *D. suzukii* to food sources and oviposition sites.^13^^,^^23^^,^^26^^,^^27^ Although there have been an increasing number of studies attempting to identify which specific compounds are responsible for this attraction, the sheer number of potential compound combinations and variations in experimental set-up have made this task difficult. Some studies focus on highly abundant compounds emitted by fruits or fermentations, or only those that elicit high antennal responses without examining the actual behavioral response (reviewed in a study by Cloonan et al.^23^). However, these approaches do not take into account the complexity of aroma perception or the potential interaction between aroma compounds in complex natural settings. Work from Cha and colleagues identified 4 components from wine and vinegar (acetic acid, ethanol, acetoin, and methionol)^28^ that, when combined into a synthetic lure, could increase trap selectivity for *D. suzukii*,^29^^,^^30^ including in natural settings, although the effect on non-target flies appeared to be environment-dependent.^31^

To tackle the challenge of screening and identifying (combinations of) potential fruit fly attractants, we used a different approach and tapped into an established set of naturally occurring, complex aroma matrices to screen for potential fruit fly attractants. Fermented beverages, such as beer, often encompass innumerable combinations of hundreds of different compounds in varying concentrations that likely resemble the mixtures of plant- and microbe-derived compounds that fruit flies encounter in their natural niches.^32^ In fact, beer is often used as an efficient insect lure in home-made traps for wasps, bees, snails, beetles, and fruit flies.^33^ Several studies have used simple yeast fermentations to develop new chemical lures.^24^^,^^34^ Moreover, utilizing a combination of compounds can more effectively attract flies compared to single compounds alone.^35^^,^^36^ However, so far, these approaches fail to grasp the true complexity of natural odors, do not focus on developing more specific traps, nor do they shed light on the causes for observed differences in fly behavior for specific compounds. Using a combination of laboratory assays and greenhouse trials, we set out to (1) identify different compounds in beers that act as fly attractants; (2) determine if differences in odor-evoked neuronal responses in the fly brain reflect the observed differential olfactory preferences of *D. melanogaster* compared to *D. suzukii*; and (3) investigate if different (subsets and combinations of naturally occurring) compounds could be used to enhance the specificity of currently used traps.

We recently performed a detailed chemical analysis of 250 different Belgian beers, measuring over 80 different compounds and chemical properties associated with fermentation.^37^^,^^38^ From this set, we selected 45 beers that differ significantly in their aroma, and directly tested their attractiveness to *D. suzukii* and its close relative *D. melanogaster* using laboratory trap-based assays. We found that the two species differ in their preferences for the tested beers and beer styles. Using a multivariate statistical approach, we linked fly preferences to our catalog of beer aroma profiles to predict potentially attractive compounds. Screening these individual compounds at concentrations found in beer, using laboratory trap-based assays, demonstrated species-specific behavior. Next, we showed that different (ecologically relevant) compounds result in different odor-induced activity patterns in the antennal lobes of female flies, providing additional evidence for the notion that major changes in olfaction have accompanied the evolution of *D. suzukii*’s attraction to ripening fruits. Finally, we used these insights to examine whether these compounds could enhance the specificity of an existing commercial synthetic chemical lure. We demonstrate that use of linalool, geraniol, β-cyclocitral, and dimethyl sulfide (DMS) could increase the selectivity of commercial lures for *D. suzukii* over *D. melanogaster*. We also show that, individually, some of these compounds enhance sex-specific *D. suzukii* attraction to lures under greenhouse conditions. These experiments provide direct insight into what compounds or mixture of compounds could be more effective at trapping and monitoring *D. suzukii* in the field, contributing to overall better monitoring and management of this important pest species.

## Results

### *D. suzukii* and *D. melanogaster* have distinct beer preferences

**Figure 1:**
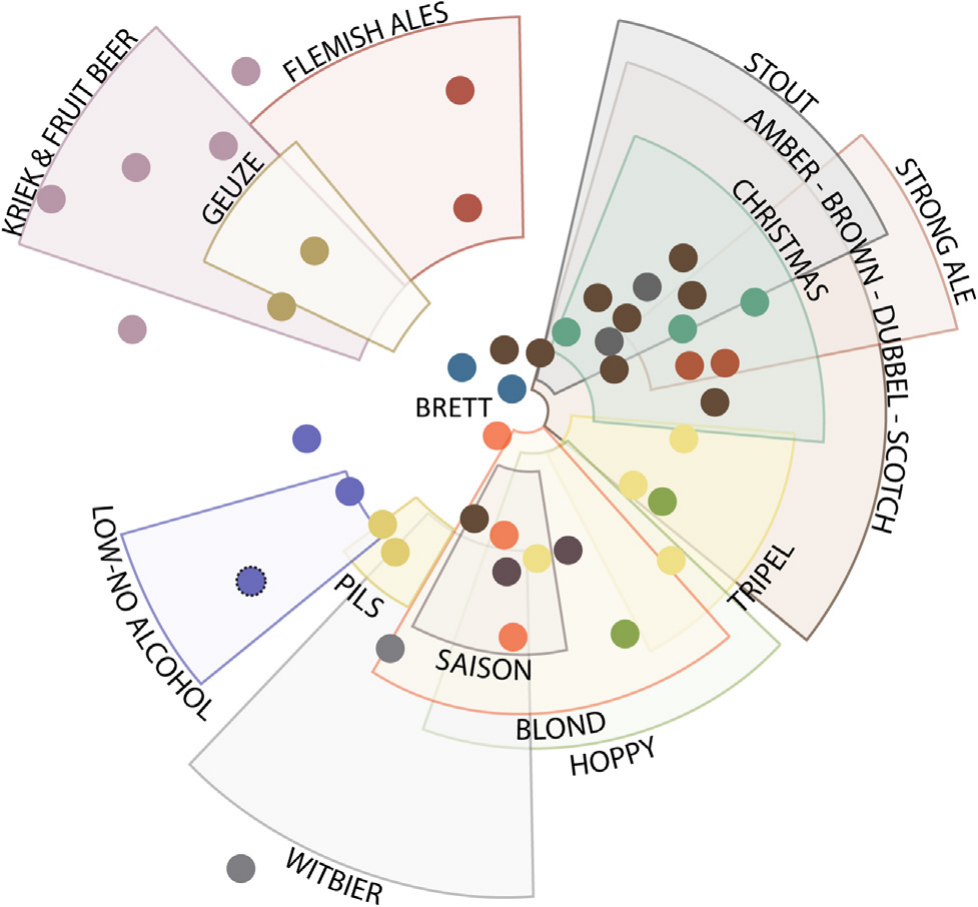
Selection of beers for preference assays The “beer map” depicts the distribution of classic beer styles with beers positioned based on chemical characteristics (adapted from a study by Roncoroni and Verstrepen^37^). Beers that are close to each other on the map have similar aroma profiles. The relative positions of the 45 selected beers are shown, color coded by style (full list of beers in Table S1). ∗Piedbœuf Blond is used as the “blank” beer throughout the various assays (indicated with a dotted outline on the map in the low-no alcohol section), since this beer had relatively low levels of all aroma compounds measured. See also Table S1.

**Figure 2:**
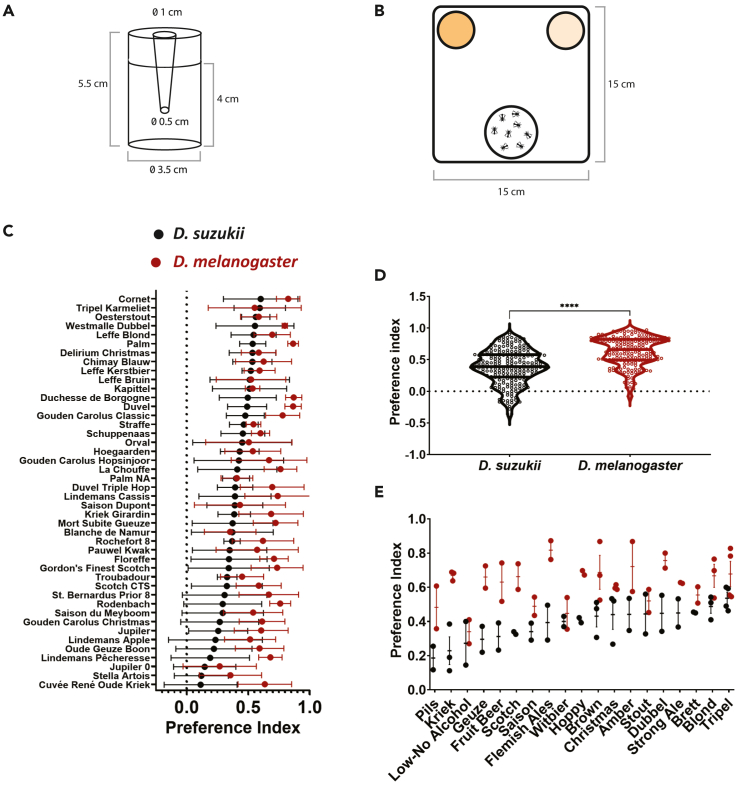
*D. suzukii* and *D. melanogaster* have distinct beer preferences (A) Traps were constructed using polystyrene containers and polypropylene lids cut to the dimensions indicated. (B) Top view of arrangement of traps in the small arena to measure beer preference indices. Differently colored traps represent different beers tested in the arena. (C) Preference indices (defined as $\frac{n{}^{\circ}\;flies\;in\;beer-n{}^{\circ}\;flies\;in\;blank}{total\;n{}^{\circ}\;flies\;caught\;\left(beer+blank\right)}$ ) for each tested beer, ranked by increasing average of *D. suzukii* preferences (black). *D. melanogaster* indices for the corresponding beers are shown in red. Values shown are mean ± SD, *n* = 4–8 for each beer. (D) Overall preference for beers by species. *D. suzukii* preferences averaged 0.3850 vs. *D. melanogaster* at 0.6281. Each dot represents the preference index of one measurement for one specific beer, with each beer tested at least 4 times. To test for significant differences, we used a Welch’s t test. ∗∗∗∗*p* < 0.0001. (E) Preference indices by beer style category, ranked by increasing average for *D. suzukii.* Dots represent average for a specific beer within a beer style category, bar is mean ± SEM per beer style. See Table S1 for division of beers tested in different beer styles. Raw data, including numbers of captured flies and preference indices, are available on Mendeley Data (see STAR Methods for more details). See also Figures S1, S2, and Table S1.

A selection of 45 beers (at least two beers from each beer style as defined in studies by Roncoroni and Verstrepen^37^ and Schreurs et al.^38^; with selected beers covering the diverse beer aroma landscape, see Figure 1; Table S1) was tested for attraction of both *D. suzukii* and *D. melanogaster* using a trap-based assay (Figures 2A and 2B, see STAR Methods for details). Prior to each assay, *D. suzukii* and *D. melanogaster* were starved using starvation times that yielded sufficiently high response rates and survival rates for both species (see STAR Methods and Figure S1).

Preference indices (defined as $\frac{n{}^{\circ}\;flies\;in\;beer-n{}^{\circ}\;flies\;in\;blank}{total\;n{}^{\circ}\;flies\;caught\;\left(beer+blank\right)}$ ) were calculated for both species for individual beers (Figure 2C). In general, *D. melanogaster* showed overall higher preference for beers compared to *D. suzukii* (Figure 2D, *p* < 0.0001, Welch’s t test)*. D. melanogaster* also showed a higher average response rate, but response rate was not indicative of preference index (Figure S2A, simple linear regression, R^2^ = 0.07 for *D. suzukii* and R^2^ = 0.002 for *D. melanogaster*). For both fruit fly species, preference indices for the different beers are quite well correlated between males and females (Figures S2B and S2C, simple linear regression, R^2^ = 0.534 for *D. suzukii* and R^2^ = 0.649 for *D. melanogaster*, respectively).

Interestingly, preference patterns differed between the two species; the most preferred beer of *D. suzukii* (Cornet) was not the most preferred of *D. melanogaster* (Duchesse de Bourgogne), nor was this the case for the least preferred (Cuvée Rene Oude Kriek vs. Jupiler 0.0, respectively). More generally, the two fly species seem to prefer different beer styles (Figure 2E). *D. suzukii* seems to prefer tripels and blonds, while *D. melanogaster* seems to prefer darker beers like browns, ambers, dubbels, and Flemish ales. Tripels and blonds fall more in the spicy and hoppy end of the beer aroma spectrum, whereas browns and dubbels tend to be associated more heavily with yeast and malt aromas.^37^^,^^38^ Flemish ales (along with fruit-based beers, krieks, and geuze) have additional characteristics, namely acids and phenolic compounds, arising from wild flora such as bacteria and non-*Saccharomyces* yeasts.^39^ This difference in preference could potentially reflect the natural preference of *D. melanogaster* to the aromas associated with rotten, fermenting fruits and of *D. suzukii* for plant-derived aromas.

### Multivariate analysis reveals potentially causative compounds for *Drosophila* behavioral responses

**Figure 3:**
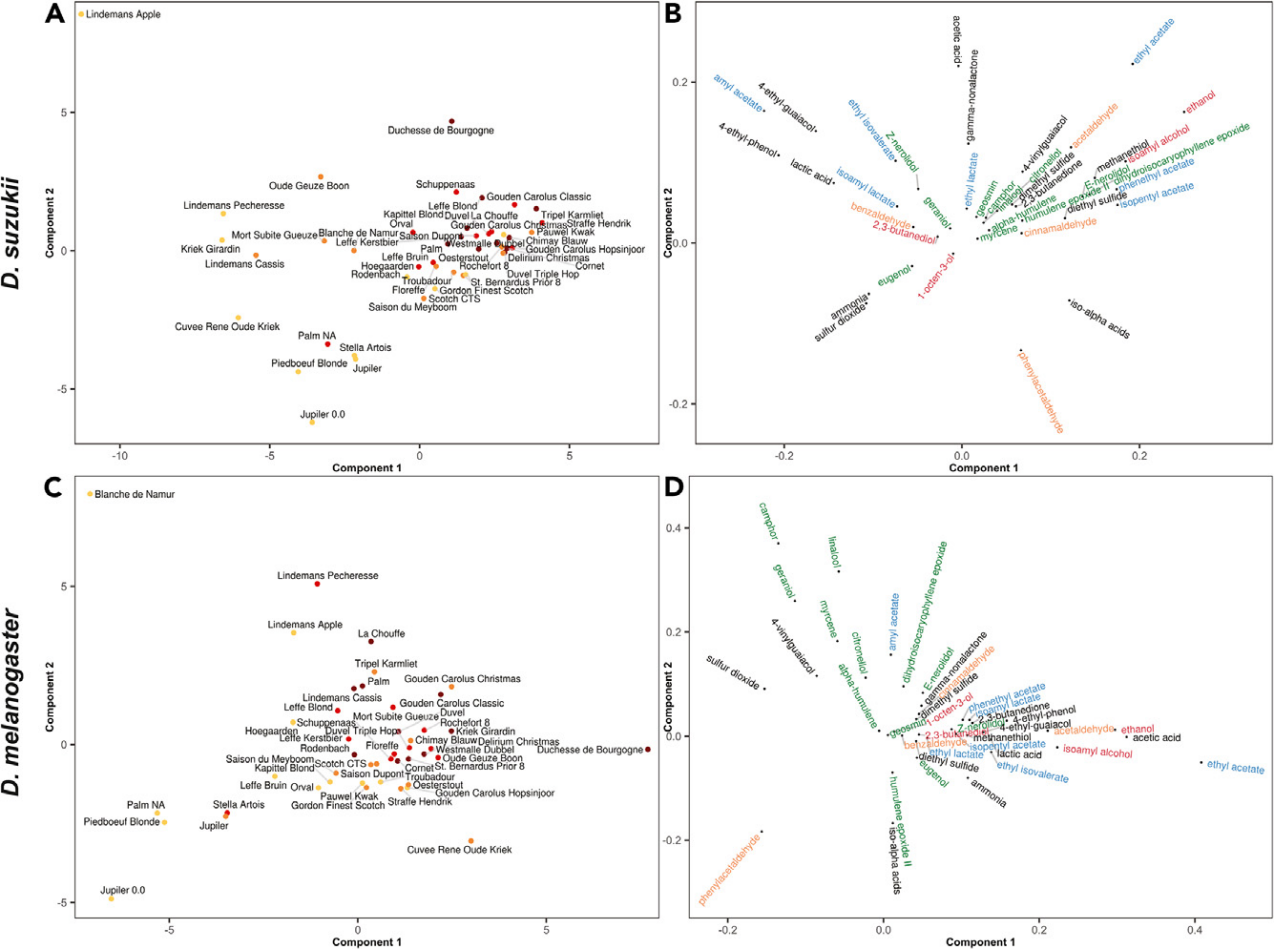
Multivariate analysis of beer composition and *Drosophila* preference indices Loadings plots show compound distributions along the first two Partial Least Squares (PLS) components for (A and B) *D. suzukii* and (C and D) *D. melanogaster.* Labels are colored by compound type (green = terpenoid, blue = esters, red = alcohols, orange = aldehydes, black = misc.). Only compounds individually tested in subsequent experiments are displayed for easier interpretation (complete loading plot shown in Figure S3). See also Table S2 for the origin (hops, spices, yeast, malt) of the different compounds in beer.

The distinct style preferences, and thus likely distinct chemically based, aroma preferences, of *D. suzukii* and *D. melanogaster* suggest that the most prominent attractive compounds might be revealed by correlating the chemical composition of each beer to the preference score for each fly species. We employed partial least-squares regression to dissect which beer-related compounds may be responsible for attraction of the flies (Figures 3 and S3). This type of analysis is particularly suited for identifying latent combinations of compounds that may be most influential in determining fly preference indices, while maintaining high statistical and predictive power in the presence of high collinearity between predictors.^40^^,^^41^

Candidate compounds were identified by their loading weights, as outlined in a study by Mehmood et al.^42^ In general, hop-related compounds (terpenoids, indicated in green in Figure 3) were more strongly associated with *D. suzukii* preference than *D. melanogaster* preference. This compound preference is also in line with the observed beer style preferences for the two species (Figure 2).

### Specific beer compounds can selectively attract *Drosophila* species

**Figure 4:**
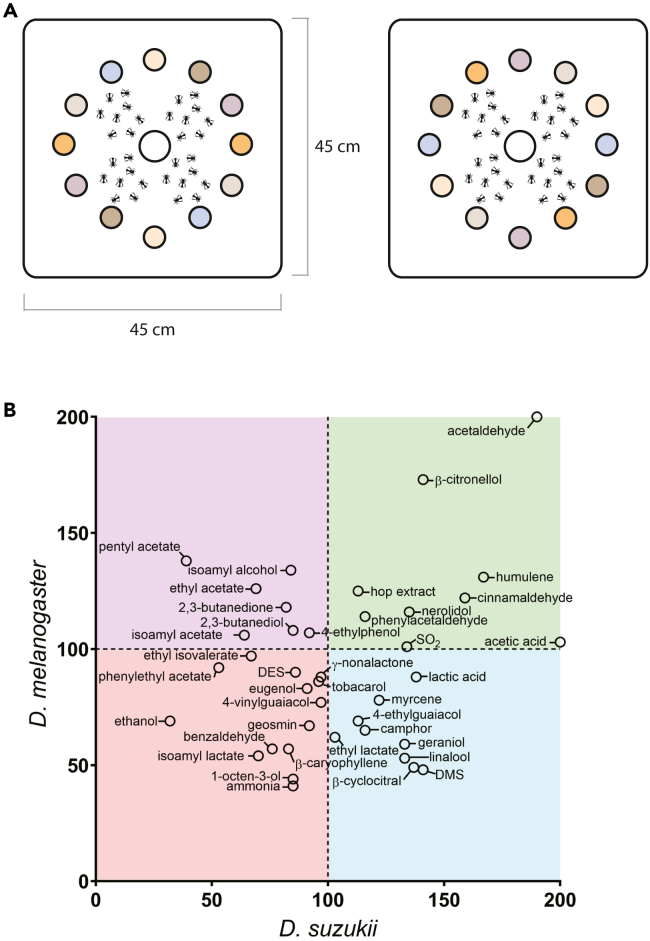
*Drosophila suzukii* and *Drosophila melanogaster* are differentially attracted to key beer-related compounds (A) Top view of arrangement of traps in the large arena to identify attractive compounds. Compounds were spiked in blank beer and each compound (plus a non-spiked blank beer) was tested twice in each arena in opposite locations (as indicated by trap coloring) and in a subsequent round, each compound was repositioned (right panel), resulting in each compound being tested 4 times. Traps used are depicted in Figure 2A. (B) Both *Drosophila* species were subjected to a trap-based compound screen to determine relative attractiveness or repellence of selected beer compounds. Values shown are averages across arenas of normalized fly catch within one arena (relative to the unspiked control beer), for more details: see STAR Methods. Quadrants are colored to more clearly depict which regions could be attractive for both (green), *D. suzukii* only (blue), *D. melanogaster* only (purple), or neither (red); with a values above 100 indicating potentially attractive compounds, and values below 100 indicating potentially repellent compounds. Note: some data points were too high to clearly position on this scale and are shown at the edge of the graph. Normalized values for out of range data points: acetaldehyde = 190 *D. suzukii*, 529 *D. melanogaster*; acetic acid = 257 *D. suzukii*, 103 *D. melanogaster*. See also Table S3. Raw data, including numbers of captured flies, are available on Mendeley Data (see STAR Methods for more details). See also Figures S4 and S5.

As many of these compounds are metabolically connected, it is possible that the observed correlations could be confounded by, or an artifact of, other variations. We, therefore, next selected several compounds that were associated with different preference indices for the different species and that have different ecological origins to individually screen for potential attraction or repellency of each species. Compounds were tested at concentrations corresponding to the highest concentration detected in beer^37^ (see Table S2 for a list of compounds and the concentrations tested). All compounds were commercially available, except for the hop-related compound tobacarol, which we chemically synthesized ourselves (see also Figure S4). Compounds were spiked into blank beer (Piedboeuf) and tested using traps arranged inside a 45 × 45 × 45 cm arena (Figure 4A), based on a previously published compound screen assay.^43^ Pilot assays demonstrated the efficacy of the screen with a positive response (i.e., attraction) of *D. suzukii* for high preference indexed beers (Palm and Cornet) and the known attractant β-cyclocitral (Figure S5A). No major position bias was seen after all compounds had been screened (Figure S5B, one-way ANOVA with multiple comparison test, for *p* values see Table S3). The number of flies caught per species indicates that some compounds are attractive for both *D. suzukii* and *D. melanogaster*, while others appear to be more species-specific (Figure 4). Notably, acetaldehyde appeared to elicit strong attraction in both species, while ethanol appeared to be strongly repellent in our setup.

Consistent with the multivariate analysis (Figure 3), yeast-based aromas, especially esters like pentyl acetate and ethyl acetate, appeared to be more attractive for *D. melanogaster*, while plant-based compounds, such as linalool and geraniol, appeared to be more attractive for *D. suzukii*. Several of these plant compounds attracted comparable numbers of flies as a known *D. suzukii* attractant, β-cyclocitral.^44^ Apart from acetate esters, we found that also other yeast-produced aroma compounds appeared to be preferred by *D. melanogaster*, such as diacetyl (2,3-butanedione).

### Functional calcium imaging identifies glomeruli activated by selected compounds

**Figure 5:**
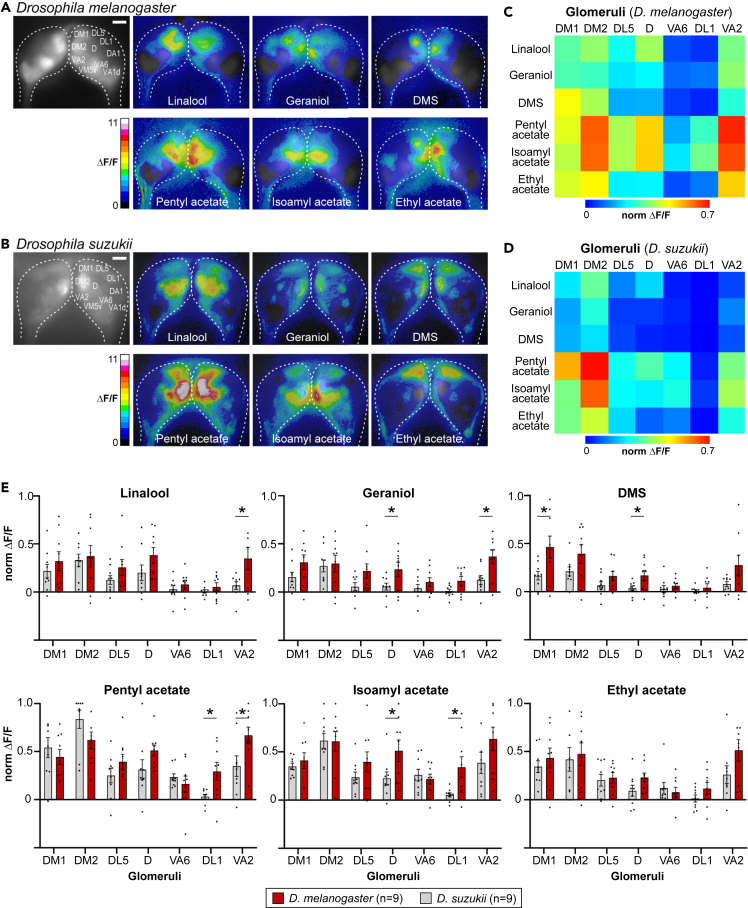
Odor-evoked calcium patterns in the antennal lobes of *Drosophila melanogaster* and *Drosophila suzukii* show species-specific responses (A and B) Representative odor-evoked calcium responses of olfactory sensory neurons in the antennal lobes of *Drosophila melanogaster* (A) and *Drosophila suzukii* (B) using transgenic flies expressing the calcium-sensitive protein GCaMP6f under control of the Orco promotor via the GAL4-UAS system. Gray-scale image (left) represents the antennal lobe structure with identified glomeruli. Calcium responses to 6 different odors (concentration: 1:10 dilution in solvent) are shown as false-color coded images scaled to the same MIN/MAX (i.e., 0–11) given by the color bar. Scale bar: 20 μm. (C and D) Functional heatmap of averaged odor-evoked calcium responses of *Drosophila melanogaster* (C) and *Drosophila suzukii* (D). The odor responses of olfactory sensory neurons for 7 glomeruli are shown for the 6 odors shown in (A) and (B) (concentration: 10^−1^). Each data point represents the averaged glomerular responses of 9 flies for each species. Responses were normalized to highest calcium response in each fly over all odors before averaging. (E) Normalized glomerular responses to 6 odors are shown as a comparison between the two species, *Drosophila melanogaster* (dark red) and *Drosophila suzukii* (gray). Bar plots show the mean (+/− SEM), black dots represent individual flies (*n* = 9 for both species). Responses were normalized to highest calcium response in each fly over all odors before averaging. Significant differences are indicated with asterisks (∗*p* < 0.05; unpaired t test). Raw data are available on Mendeley Data (see STAR Methods for more details).

**Figure 6:**
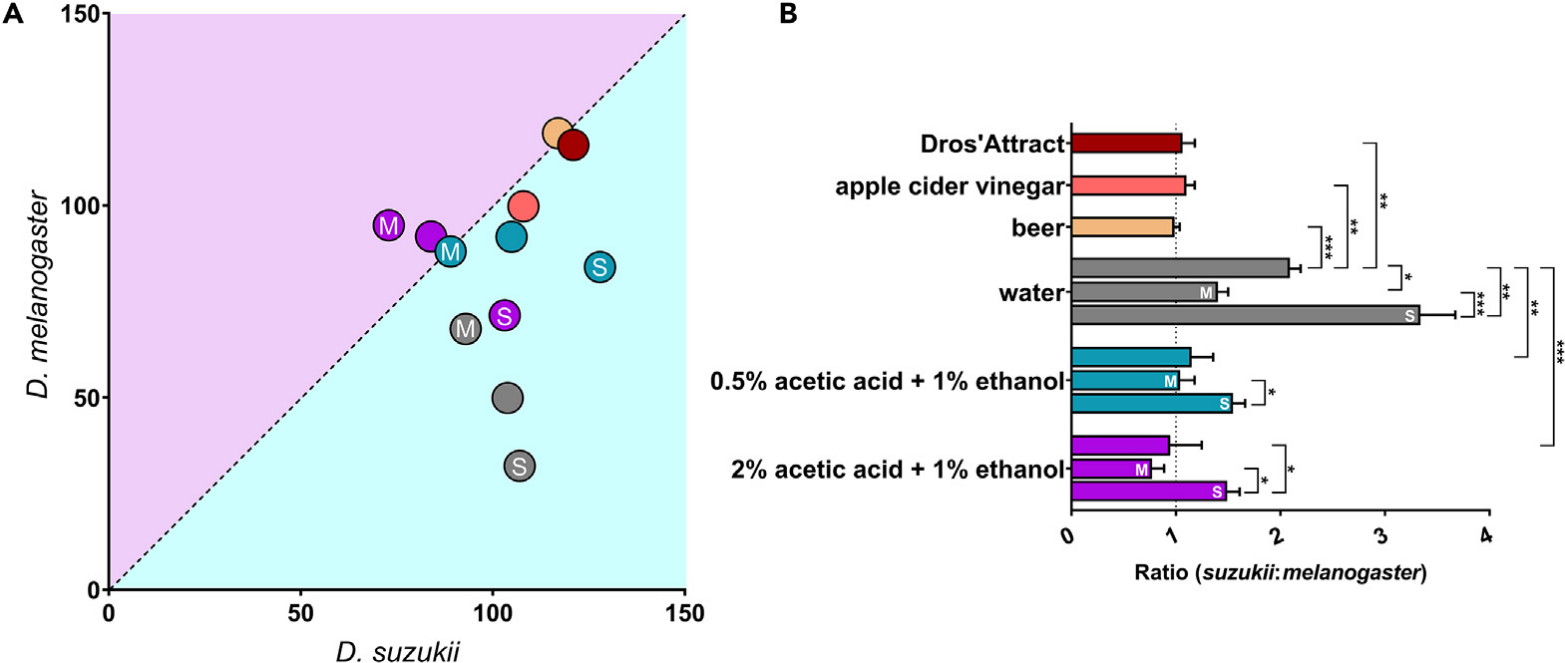
Species-selective compounds can enhance catch ratio of *D. suzukii* and *D. melanogaster* Flies caught in the Droso’Trap were sorted by species and sex, and then counted. (A) Total number of *D. suzukii* vs. *D. melanogaster* caught in the same trap using the commercially available *D. suzukii* lure Dros’Attract (red), apple cider vinegar (orange), beer (yellow), 0.5% acetic acid +1% ethanol (turquoise), or 2% acetic acid +1% ethanol (magenta). Data points with “S” contain linalool, geraniol, dimethyl sulfide, and β-cyclocitral. Data points with “M” contain pentyl acetate, ethyl acetate, isoamyl acetate, and isoamyl alcohol. (B) Ratio of *D. suzukii* to *D. melanogaster* caught in the same trap (mean ± SD, *n* = 3). Bar colors correspond to those in (A). Significance was calculated using one-way ANOVA with multiple comparisons between the background matrices and controls as well as between lures with their respective backgrounds (see Tables S4–S8 for results ANOVA analyses). ∗*p* < 0.05, ∗∗*p* < 0.01, ∗∗∗*p* < 0.0001. Raw data, including numbers of captured flies, are available on Mendeley Data (see STAR Methods for more details). See also Figure S6.

We next wondered whether the observed differences in *D. suzukii* and *D. melanogaster* preference behavior for specific odors are reflected in species-specific olfactory responses. Flies sense odorants using olfactory receptor neurons, mainly located in their antennae and their maxillary palps.^45^ Each olfactory receptor neuron usually expresses one odorant receptor (together with the co-receptor, Orco).^46^ All olfactory receptor neurons expressing the same odorant receptor converge onto the same glomerulus in the antennal lobe, the first olfactory center of the insect brain.^47^^,^^48^ It has previously been shown in *D*. *melanogaster* that a subgroup of glomeruli are responding in a valence-specific manner, meaning that they are activated by either an attractive or an aversive odor compound.^49^ Hence, the odor-evoked activities of certain glomeruli are correlated with olfactory preference in behavioral assays. We therefore aimed to investigate how the yeast- and plant-based components are represented in the antennal lobes of both *Drosophila* species and whether valence-specific glomeruli are activated. To do so, we visualized odor-induced activity patterns in the female fly antennal lobe using the GAL4-UAS system to drive selective expression of the genetically encoded calcium sensor G-CaMP6f in olfactory receptor neurons.^50^^,^^51^ While genetic tools including activity-dependent reporters are well-established for *D. melanogaster*, until recently such a reporter was not available for *D. suzukii.* We have recently generated transgenic lines for *D. suzukii* to express GCaMP6f under control of the Orco promotor via the GAL4-UAS system,^52^ enabling us to monitor odor-evoked responses selectively in olfactory sensory neurons of this species. We selected compounds that could be potentially attractive or repellent, based on our arena assays (Figure 4), also taking into account chemical diversity of the compounds. Our calcium imaging recordings show that both fly species exhibit clear and reproducible odor-specific responses to all compounds tested (Figures 5A and 5B). When comparing the odor responses between both species, we observed that the same glomeruli were activated leading to similar odor response patterns confirming our recently published study that compares the odor code in the *Drosophila* genus.^52^ However, when we consider the activity strength of specific glomeruli, clear differences are visible between *D. melanogaster* and *D. suzukii* as illustrated by a heatmap of glomerular responses (Figures 5C and 5D). Importantly, we observe significant stronger responses in glomerulus D to geraniol and DMS in *D. melanogaster*, while this glomerulus was only slightly activated in *D. suzukii* (*p* values = 0.042 for geraniol and 0.036 for DMS; Figures 5C–5E). Glomerulus D is an aversive-coding glomerulus,^49^ indicating that these compounds rather repel *D. melanogaster.* This is in line with the results from our trap-based compound screen (Figure 4B) as well as our synthetic lures (Figure 6), offering a potential explanation for the observed species-selectivity for these compounds. In other words, activation of this aversive-coding glomerulus could potentially explain why geraniol and DMS act as a repellent for *D. melanogaster*. We also observed significantly stronger responses in glomerulus VA2 to terpenes (linalool and geraniol) and fruity acetates in *D. melanogaster* (*p* values = 0.036 for linalool, 0.013 for geraniol, and 0.033 for pentyl acetate; Figures 5C–5E). This glomerulus has so far not been assigned any clear valence (attractive or aversive). Surprisingly, we also find that fruity acetates, such as isoamyl acetate, result in a stronger response in glomerulus DL1 in *D. melanogaster* (*p* value = 0.04*)*, which has been assigned an aversive valence.^49^ However, it is important to point out that the attractive-coding glomerulus DM2 is much stronger activated than glomerulus DL1 for all these esters, which indicates a positive preference for these odors in *D. melanogaster* flies. To summarize, both fly species show significantly different odor responses in some glomeruli and hence appear to perceive the tested compounds differently, potentially underlying their behavioral differences with regard to olfactory preference.

### Development of species-specific synthetic lures

The results from our arena-based assays indicate that we could potentially enhance species-selectivity of existing chemical lures by adding species-specific compounds. We thus created different chemical lures aimed at attracting more *D. melanogaster* or more *D. suzukii* (compared to *D. suzukii or D. melanogaster*, respectively) and tested them using competition assays with commercially available Droso’Traps (Biobest) placed in the 45 × 45 × 45 cm arena. Specifically, we combined ethyl acetate, pentyl acetate, isoamyl alcohol, and isoamyl acetate (Lure-M) as well as linalool, geraniol, DMS, and β-cyclocitral (Lure-S), because of their chemical diversity and since our previous experiments showed that some of these compounds could have a species-specific effect, with individual compounds in Lure-M more specific to *D. melanogaster* and individual compounds in Lure-S more specific to *D. suzukii* (Figure 4). We focused on species-specific compounds since these could allow to, for example, develop more selective monitoring traps, which trap less non-target insects.

Since the background matrix can affect the perception of compounds,^32^^,^^53^ various matrices were tested. Several studies utilized a mixture of acetic acid and ethanol at relatively high concentrations (1–4% and 7–8%, respectively).^28^^,^^43^^,^^54^^,^^55^ However, we observed that ethanol can reduce the numbers of flies caught, and that *D. suzukii* appears to be attracted to low levels of acetic acid, while *D. melanogaster* is not (Figure 6). Therefore, we used lower levels of both compounds in two different combinations: 0.5% acetic +1% ethanol and 2% acetic acid +1% ethanol (both with 0.01% Triton X-100 as drowning solution). We compared our lures to “blank” beer, apple cider vinegar, and the commercially available *D. suzukii* lure Dros’Attract (Biobest, consisting of apple cider vinegar, grape must, and sugar).

Apple cider vinegar, blank beer, and Dros’Attract each caught more than half of the released *D. suzukii* but also caught almost equal numbers of *D. melanogaster* (Figure 6). Lure-M compounds in either synthetic background caught more *D. melanogaster* than *D. suzukii*. Lure-S compounds enhanced *D. suzukii*-specificity compared to Lure-M compounds as well as compared to the matrix, with the 0.5% acetic acid background being slightly more effective (for *p* values of all comparisons using one-way ANOVA, see Tables S4 and S5). This combination caught the same number of *D. suzukii* individuals as the commercial Dros’Attract, but with a 30% reduction in *D. melanogaster*. Surprisingly, Lure-S compounds added to just water was the most selective lure tested. Although 14% fewer *D. suzukii* were caught compared to Dros’Attract, Lure-S in water reduced *D. melanogaster* catches by 75%. Importantly, the observed increase in selectivity for *D. suzukii* when using Lure-S compounds did not affect the overall attractiveness to *D. suzukii*, and this holds for all background matrices tested (one-way ANOVA with Tukey’s multiple comparison test; *p* values can be found in Table S6).

At first glance, Lure-M compounds did not appear to significantly enhance *D. melanogaster*-specificity. However, this is likely due to differing sex-specific behaviors of the flies when using the acetic acid + ethanol background matrices (Figure S6). For example, 2% acetic acid +1% ethanol alone caught similar numbers of male and female *D. melanogaster* (and *D. suzukii*). Adding Lure-M compounds greatly increased the number of male *D. melanogaster* caught and reduced the number of male *D. suzukii*. However, this had the opposite effect on females; fewer *D. melanogaster* females were caught with slightly more *D. suzukii* females. A similar effect can be seen with Lure-S compounds in 0.5% acetic acid +1% ethanol; more than double the number of *D. suzukii* females are caught compared to *D. melanogaster* with equal numbers of males from both species. This sex-specific effect is less dramatic when using water as a background; the addition of Lure-S compounds enhances attraction of *D. suzukii* females without significantly affecting males (for *p* values of all comparisons using one-way ANOVA, see Tables S7 and S8).

### Some Lure-S compounds improve commercial lures in a sex-specific manner

**Figure 7:**
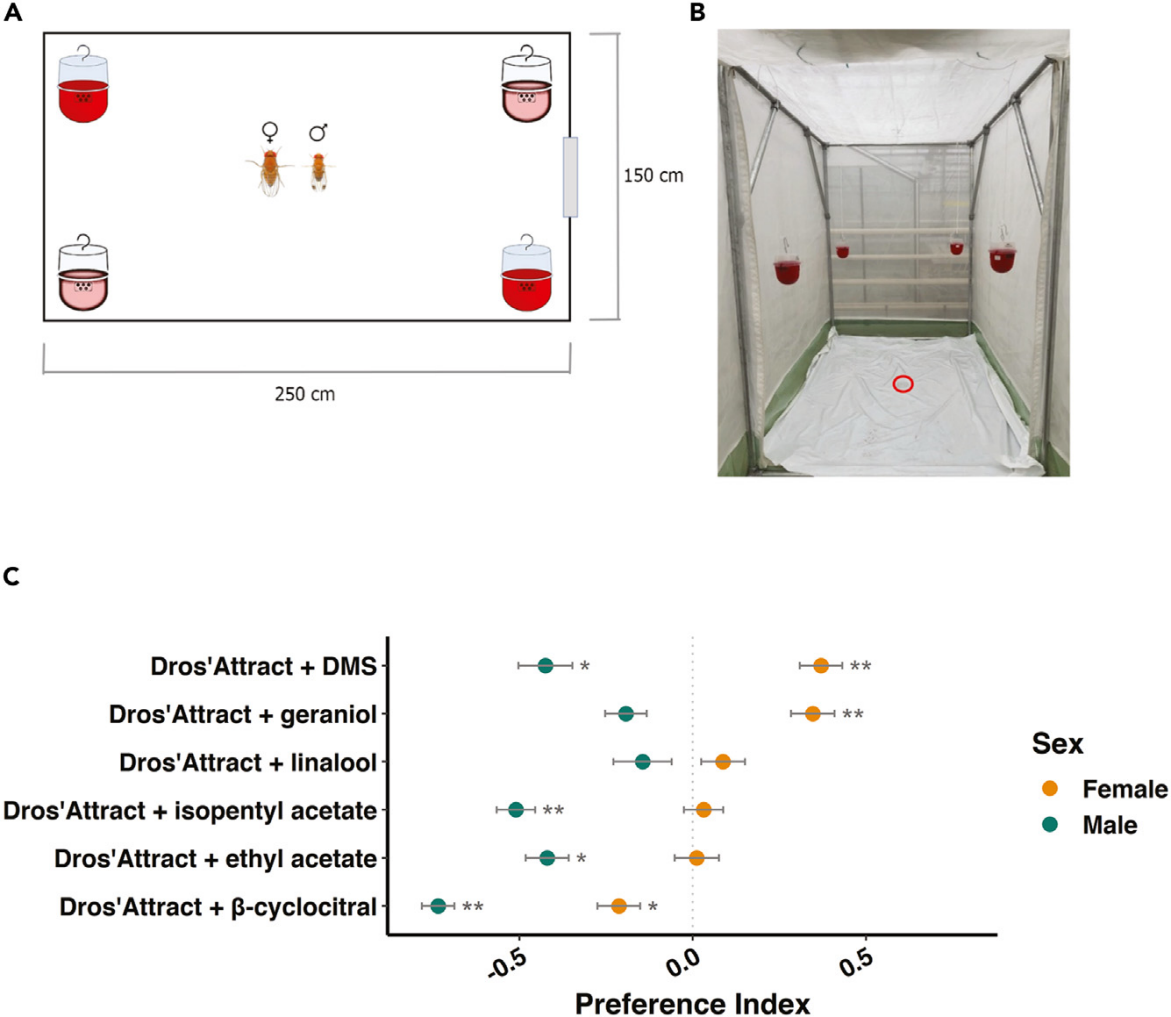
Candidate compounds increase female *D. suzukii* attraction to a commercial lure Diagram (A) and photograph (B) of the experimental setup for greenhouse experiments. 250 × 150 × 200 cm arenas were placed in a greenhouse compartment with tomato plants (*Solanum lycoperscium*). Each arena contained four Droso’Traps, with two controls containing Dros’Attract (pink) and two treatments containing Dros’Attract spiked with a single compound (pink). 25 male and 25 female *D. suzukii* were released at ground level from the middle of the arena, indicated by a red circle in the photograph. The experiment was repeated 15 times per compound, with differing trap arrangements to eliminate position bias. (C) Preference indices of male (green) and female (orange) *D. suzukii* in response to a commercial lure (Dros’Attract) spiked with a single Lure-S compound. Values shown are mean ± SEM, *n* = 15 for each compound and sex. Tests for significant differences between control and treatment groups were performed using the Wilcoxon signed-rank test, with the Holm-Bonferroni method for multiple corrections. ∗*p* < 0.05, ∗∗*p* < 0.01. Raw data, including numbers of captured flies and preference indices, are available on Mendeley Data (see STAR Methods for more details).

To further test compounds’ effectiveness in enhancing *D. suzukii*’s attraction to an existing commercial lure, individual Lure-S compounds were added to Dros’Attract and traps were placed under greenhouse conditions (Figures 7A and 7B, see also STAR Methods for more details). This allowed us to study more natural flight responses of *Drosophila*, compared to the smaller trap assays used in previous set-ups. DMS and geraniol were found to significantly enhance *D. suzukii* females’ attraction when added to Dros’Attract (Figure 7C, *p* value = 0.008 for DMS, *p* value = 0.008 for geraniol, Wilcoxon signed-rank test, with Holm-Bonferroni method for multiple corrections), while linalool had no effect (*p* value = 0.491). In contrast, *D. suzukii* males generally reacted aversely to these compounds. Somewhat surprisingly, β-cyclocitral, reported in literature and confirmed by our previous assays to selectively attract *D. suzukii*, repelled both sexes when added to Dros’Attract (*p* value = 0.034 for males and 0.008 for females). This has also previously been observed in other studies, with β-cyclocitral reducing attraction to an otherwise effective bait when deployed in blueberry, blackberry, cherry, and raspberry orchards, despite attracting *D. suzukii* in single-compound tests under laboratory settings.^22^ This further underscores the importance of matrix/background effects, as well as the specific experimental and environmental conditions used.

Similarly, the Lure-M compounds ethyl acetate and isoamyl acetate were individually tested on *D. suzukii* under greenhouse conditions, yielding no significant effects on females and strong repellent effects on the males. This agrees with our previous assays, further indicating that these yeast compounds are unattractive to the pest species (Figure 4).

## Discussion

There is increasing evidence that aroma-mediated interactions between plants, microbes, and insects represent important components of insect ecology. However, much of the ongoing research focuses on the response of one specific (model) insect species to one or a few pure aroma compounds. While this approach offers insight into the nature and molecular mechanisms of the response, it actually is an inaccurate representation of natural settings where insects are confronted with complex odor blends, a multitude of sensory signals as well as other species.^25^ It is therefore perhaps not surprising that some of the most effective and most commonly used insect lures are still natural fermentation products like beer or cider which contain hundreds of ecologically relevant plant and microbial metabolites. The downside of this approach is that these complex natural mixtures often attract a wide variety of species, including both target and non-target species. By contrast, many commercial lures (such as e.g., Dros’Attract) only utilize a small subset of the most prevalent chemicals such as ethanol or acetic acid. Although some synthetic lures are starting to explore other compounds,^29^ it is likely that other aroma compounds present in fermentations could play a role in insect attraction and have the potential of being more potent or species-specific.

In this study, we combined detailed chemical analyses of complex fermentations with multivariate statistical approaches and behavioral assays to identify compounds that could specifically attract or deter the agricultural pest *D. suzukii.* To this end, we directly compared the behavioral responses of *D. suzukii* to that of the closely related (and harmless) common fruit fly *D. melanogaster* to assess potential differences. Starting from carefully selected candidate beers with distinct aroma profiles, together with our extensive measurements of distinct beer compounds^37^^,^^38^ and individual compounds testing, we were able to identify potential species-selective compounds. Our results indicate that fruity yeast-derived esters more specifically attract *D. melanogaster*, whereas plant-derived volatiles like linalool, geraniol, camphor, and myrcene are more specific for *D. suzukii*.

Interestingly, some compounds, including previously identified attractants, did not yield the expected behavioral response. For example, *D. melanogaster* is sometimes referred to as the “vinegar” fly due to its attraction to vinegar (acetic acid). In our setup, (relatively low levels of) acetic acid proved to be attractive for *D. suzukii*, but less so for *D. melanogaster.* Household vinegar typically contains 5% acetic acid whereas the amount tested in our screen was only 0.5%–2%, reflective of concentrations found in some beer. This highlights the importance of dose-dependent behavioral responses. Additionally, apple cider vinegar was able to catch equal numbers of both species while reduced acetic acid levels were more effective at catching *D. suzukii*. The synthetic lure matrices also demonstrate that decreasing acetic acid levels shifts the species ratio toward *D. suzukii.* Our results therefore indicate that *D. suzukii* may be more sensitive to lower levels of acetic acid compared to *D. melanogaster,* and thus using lower acetic acid levels could enhance species specificity of lures.

This dose-dependency is not unique^54^ and in fact becomes more complex when it comes to trying to predict insect behavioral responses. For example, β-cyclocitral, a well-known *D. suzukii* attractant,^44^ becomes repellent with a 10-fold increase in the concentration (Figure S5). Other plant-based attractants identified here, including linalool, are more commonly considered insect repellents in studies where they are typically tested undiluted or at high concentrations.^56^ This suggests that insect behavior may change from repellence to attraction or vice versa for certain compounds when the concentration diminishes over time or diffuses across larger distances. Therefore, as experiments are often carried out in controlled, closed environments, the design of any effective lure should include a careful testing of concentrations and distance effects in addition to testing different compounds at a single concentration.^57^ Furthermore, background odors and visual cues could have a significant effect on insect orientation and responses.^58^ Relative attraction to a specific lure also depends on the physiological state of the fly.^59^ Growing season^24^ and even the type of trap used^60^ have been described as relevant factors influencing insect preference.

Testing the effects of a selected number of plant and yeast compounds when added to an existing commercial lure deployed among crop plants led to results that differed somewhat from the experiments performed in laboratory settings. For example, linalool and β-cyclocitral, previously demonstrated to enhance attraction in beer, had no or repellent effects respectively when applied to Dros’Attract, an existing commercial lure, under greenhouse conditions. Nonetheless, for other compounds, similar behaviors were found in both lab and greenhouse settings, with geraniol and DMS enhancing *D. suzukii* females’ attraction, while the yeast compounds ethyl acetate and isoamyl acetate had no or repellent effects on both sexes.

Our results suggest that it is possible to deploy geraniol and DMS to improve lure performance in natural settings. Our results also demonstrate the feasibility of deriving multiple causative compounds for attraction, using multivariate statistics combined with behavioral assays in response to a complex mixture such as beer. Also from an applied point of view, improving the attractiveness and/or specificity of commercial attractants is of great interest and opens up opportunities for further development of *D. suzukii* attractants. Overall, our data not only indicate that selected compounds can enhance current lures, but also suggest an alternative path to draw *D. suzukii* away from target fruits. Planting plant species that produce high levels of such attractive compounds such as laurel, rosemary, saffron, sage, and thyme nearby could potentially deter *D. suzukii* from the fruits and concentrate them on these trap plants where they can be locally treated or caught.^61^ In addition to placing traps near host plants, traps placed near these plants could help increase numbers of *D. suzukii* caught and reduce overall population levels.

In addition to providing insight into improving lure efficacy and specificity, our results also further our understanding of the importance of olfactory cues for the ecology of both flies. Previous studies have already identified several important sensory cues used by *D. suzukii* in host searching,^12^^,^^13^^,^^14^ revealing that *D. suzukii* differs from *D. melanogaster* in how it senses bitter compounds and sugars as well as in mechanosensing. Our study now further expands these results and identifies other important olfactory cues used by different *Drosophila* species, which also matches their adaptation to different niches. Specifically, our finding that *D. suzukii* is attracted to aroma’s associated with plants and unripe fruit agrees with its preference to lay eggs in these substrates, whereas the attraction of *D. melanogaster* to microbial fermentation-derived fruity esters (and other by-products of alcoholic fermentation, such as 2,3-butanedione) agrees with its oviposition preference in rotting or overripe fruits. Microbial fermentation is indicative of rotting fruit, typically found on the ground around the host plant. In contrast, plant compounds will emanate directly from the host plant, both from the ripening fruit and the leaves,^25^^,^^62^ drawing *D. suzukii* toward ripening fruit despite the fact that they are also attracted to rotting fruit.^13^ *D. suzukii* adults are also less resistant to alcohol,^63^ so attraction to ripening fruits could also protect *D. suzukii* flies from toxic alcohol levels in fermenting fruits.

Our calcium imaging results show that compounds identified as being less attractive to *D. melanogaster* in our assays (geraniol and DMS) elicit a significantly stronger response in glomerulus D, which is mostly activated by aversive odors. *D. suzukii* shares a last common ancestor with *D. melanogaster* ∼15 million years ago^64^^,^^65^; and our results further support the notion that major changes in olfaction have accompanied evolution of attraction of *D. suzukii* to ripening fruit.

The wide accessibility of beers makes our findings especially relevant for the general public. Synthetic compound mixtures are not always available for commercial use and they can be economically unfeasible for home use. Beer has been used as an efficient attract-and-kill solution for wasps, and wine is an effective way to remove *D. melanogaster* from the kitchen. This could potentially be a way to involve the public in actively monitoring *D. suzukii* and provide knowledge to optimize traps for emerging pests in the future. Altogether, our study provides a strong lead toward the development of enhanced monitoring and trapping systems for both commercial and home use.

### Limitations of the study

We identified odor-specific activation patterns of specific glomeruli in *D. suzukii* and *D. melanogaster*. Further research is needed to link the species-specific activation patterns in the brain to the observed differences in behavioral response to the tested odors.

While we demonstrated that specific compounds can indeed increase *D. suzukii* attraction to a commercial lure in a greenhouse setting, it remains to be investigated if this would also be the case for lures placed in even more complex and open settings, such as orchards and vineyards. This would also allow to more precisely determine the species-selectivity of the lures for different insect species (not only *D. suzukii* and *D. melanogaster*) by investigating the number of non-target insects caught in the traps with different lures.

## Resource availability

### Lead contact

Further information and requests for resources and reagents should be directed to and will be fulfilled by the lead contact, Kevin J. Verstrepen (kevin.verstrepen@kuleuven.be).

### Materials availability

This study did not generate new unique reagents.

### Data and code availability

• This study did not generate unique “standardized data types” or original code.
• Raw data supporting the findings of this study have been deposited at Mendeley Data and are publicly available as of the date of publication. DOIs are listed in the key resources table.
• Scripts used to analyze these date have also been deposited at Mendeley Data and are publicly available as of the date of publication. DOIs are listed in the key resources table.
• Any additional information required to reanalyze the data reported in this paper is available from the lead contact upon request.

## Acknowledgments

We would like to thank Dries De Vadder for help in creating the graphical abstract. Research in the lab of K.J.V. is supported by 10.13039/501100004040KU Leuven, VIB (10.13039/501100004727Vlaams Instituut voor Biotechnologie), 10.13039/501100003130FWO, and 10.13039/100012331Agentschap Innoveren en Ondernemen (VLAIO). Mass spectrometry was made possible by the support of the Hercules Foundation of the Flemish Government (grant 20100225–7). Research in the lab of B.L. is supported by 10.13039/501100003130FWO and 10.13039/100012331VLAIO. Research in the lab of T.W. is supported by 10.13039/501100003130FWO (G.0A51.15) and 10.13039/100012331VLAIO (HBC.2017.0820). This project has received funding from the European Union’s Horizon 2020 research and innovation programme under the Marie Skłodowska -Curie grant agreement no. 722642 (INTERFUTURE) (to F.W. and A.A.). S.A., B.S.H., and S.S. were funded by the 10.13039/501100004189Max Planck Society and the Max Planck Center “next Generation Insect Chemical Ecology (nGICE)”. The funders had no role in study design, data collection and analysis, decision to publish, or preparation of the manuscript.

## Author contributions

Experimental work: M.C.D., S.A., A.A., F.W., T.O., W.D., B.H.-M., and M.R.; methodology and design of experiments: M.C.D., S.P., B.L., S.S., and K.J.V.; data analysis: M.C.D., S.P., S.A., L.C., C.V., T.W., K.V., B.S.H., and S.S.; data visualization: M.C.D., S.P., S.A., and K.V.; writing – original draft: M.C.D., S.P., K.V., S.S., and K.J.V.; writing – review and editing: all authors; supervision: B.S.H., S.S., and K.J.V; funding acquisition: B.L., T.W., F.W., A.A., B.S.H, S.S., and K.J.V.

## Declaration of interests

F.W. is employed by Biobest, a producer of commercial insect traps.

## STAR★Methods

### Key resources table

**Table:** 

REAGENT or RESOURCE	SOURCE	IDENTIFIER
**Chemicals, peptides, and recombinant proteins**		

1-octen-3-ol	Sigma-Aldrich	W280518; CAS: 3391-86-4
2,3-butanediol	Sigma-Aldrich	B84904; CAS: 513-85-9
2,3-butanedione	Sigma-Aldrich	B85307; CAS: 431-03-8
4-ethyl-guaiacol	Sigma-Aldrich	W243604; CAS: 2785-89-9
4-ethyl-phenol	Sigma-Aldrich	W315605; CAS: 123-07-9
4-vinylguaiacol	Sigma-Aldrich	W267511; CAS: 7786-61-0
acetaldehyde	Sigma-Aldrich	00070; CAS: 75-07-0
acetic acid	Sigma-Aldrich	W200603; CAS: 64-19-7
α-humulene	Sigma-Aldrich	12448; CAS: 6753-98-6
Ammonia	Thermo Fisher Scientific Inc.	984720; CAS: 7664-41-7
benzaldehyde	Sigma-Aldrich	418099; CAS: 100-52-7
β-caryophyllene	Sigma-Aldrich	75541; CAS: 87-44-5
β-cyclocitral	Sigma-Aldrich	W363928; CAS: 432-25-7
Camphor	Sigma-Aldrich	W526606; CAS: 76-22-2
diethyl sulfide	Sigma-Aldrich	107247; CAS: 352-93-2
dimethyl sulfide	Sigma-Aldrich	W274615; CAS: 75-18-3
Ethanol	VWR International	20821321; CAS: 64-17-5
ethyl acetate	Sigma-Aldrich	270989; CAS: 141-78-6
ethyl isovalerate	Sigma-Aldrich	71607; CAS: 108-64-5
ethyl lactate	Sigma-Aldrich	69799; CAS: 97-64-3
Eugenol	Sigma-Aldrich	E51791; CAS: 97-53-0
γ-nonalactone	Sigma-Aldrich	W278106; CAS: 104-61-0
Geosmin	Sigma-Aldrich	G5908; CAS: 16423-19-1
Geraniol	Sigma-Aldrich	48798; CAS: 106-24-1
isoamyl alcohol	Sigma-Aldrich	W205710; CAS: 123-51-3
isoamyl lactate	TCI Chemicals	L0117; CAS: 19329-89-6
isopentyl acetate	Sigma-Aldrich	306967; CAS: 123-92-2
isomerized hop extract	Brouwland bvba	#053.185.5
lactic acid	Sigma-Aldrich	W261106; CAS: 50-21-5
Linalool	Sigma-Aldrich	L2602; CAS: 78-70-6
Mineral Oil	VWR Life Science	Cat#J217; CAS 8042-47-5
Myrcene	Sigma-Aldrich	64643; CAS: 123-35-3
Nerolidol	Sigma-Aldrich	W277207; CAS: 7212-44-4
pentyl acetate	Sigma-Aldrich	66962; CAS: 628-63-7
phenylacetaldehyde	Sigma-Aldrich	W287407; CAS: 122-78-1
phenylethyl acetate	Sigma-Aldrich	73747; CAS: 103-45-7
sulfur dioxide	Sigma-Aldrich	CAS: 7446-09-5
Tobacarol	This study	N/A
*trans*-cinnamaldehyde	Sigma-Aldrich	C80687; CAS: 14371-10-9

**Critical commercial assays**		

Acetic acid system reagent	Thermo Fisher	984318
Ammonia system reagent	Thermo Fisher	984320
L-Lactic Acid system reagents	Thermo Fisher	984308
Total Sulfite (SO_2_) system reagents	Thermo Fisher	984345

**Deposited data**		

Raw and analyzed data (including scripts)	This paper, Mendeley Data	Mendeley Data: https://doi.org/10.17632/vysbvp5362.3

**Experimental models: organisms/strains**		

*D. melanogaster*	This paper	N/A
*D. suzukii*	This paper	N/A
*D. suzukii Orco-GAL4* and *UAS-GCaMP6f* transgenic line	Depetris-Chauvin et al.^51^	N/A

**Software and algorithms**		

R version 4.2.3		
FIJI (ImageJ 1.53a)	National Institutes of Health, USA	
GraphPad Prism 9.0.2	GraphPad	

**Other**		

Belgian beers	Roncoroni and Verstrepen,^36^ Schreurs et al.^37^	N/A
Drosotrap	Biobest Group NV	N/A
Dros’Attract	Biobest Group NV	N/A

### Experimental model and study participant details

#### Fly rearing for beer and compound attractiveness

Initial laboratory fly stocks were kind gifts from the laboratory of Patrik Verstreken (*D. suzukii*) and Tom Wenseleers (*D. melanogaster*) (KU Leuven). The stock of *D. suzukii* (MD01) was established from insects collected at the Research Center for Fruit Growing in Sint-Truiden, Belgium. *D. melanogaster* (MD02) was initially collected from a public park in Leuven, Belgium. Field-caught flies were used to establish lab cultures that have been affected as little as possible by long-term breeding.

Both species were raised at room temperature on a basic sugar and yeast fly food mixture (0.8% agar, 5% sugar, 8% polenta, 0.08% methyl 4-hydroxybenzoate (Sigma CAS 99-76-3); all %w/v). Flies were flipped into new vials once eggs or larvae were clearly visible. Flies were transferred after anesthetization with carbon dioxide to vials containing fresh food. Flies that were stuck in the old food vials were removed by ethanol-sterilized tweezers to prevent mold growth and mite infestations. Adult flies were disposed after two weeks post-emergence.

#### Fly rearing for calcium imaging

Flies were reared at a temperature of 25°C, following a 12:12 h light-dark cycle (LD), with a humidity level of 70%, except for *D. suzukii* strains that were grown and maintained at 22°C. All fly stocks were kept in a standard corneal agar medium comprising cornmeal (10% m/v), agar (0.4% m/v), golden syrup (12% m/v), yeast (1% m/v), propionic acid (0.25% v/v) (CAS 79-09-4, Cat#6026, Carl Roth GmbH), and Nipagin 30% (0.3% v/v) (Cat#H5501, Sigma-Aldrich). For the optimization of culture in *D. suzukii*, stocks were supplemented with smashed blueberries. We generated an *Orco-GAL4* and *UAS-GCaMP6f* transgenic line for *D. suzukii* in the lab through molecular cloning. A detailed protocol for the generation of transgenic lines is described in.^52^

A complete list of the stocks can be found in the key resources table.

### Method details

#### Beer selection and compound analysis

Beer style categories were defined as in “Belgian Beer: Tasted and Tested”^37^ and in our recently published study.^38^ At least two beers from nineteen styles were selected based on availability and distribution throughout the “beer map” (Figure 1; Table S1). Piedbœuf Blond was utilized as a “blank” beer as it has a low sensory profile.^37^ Note that although there appears to be a gap in representation in the stout category, only one beer lies in the non-overlapping region. This outlier was not available at the time of selection.

Chemical compound composition data were acquired in preparation for the book “Belgian Beer: Tasted and Tested”^37^ and for our recent study.^38^ Details on how the different compounds were measured can be found in^38^ and in Table S2.

Beers within their expiration date were purchased from commercial retailers. Samples were prepared in biological duplicates at room temperature, unless explicitly stated otherwise. Bottle pressure was measured with a manual pressure device (Steinfurth Mess-Systeme GmbH) and used to calculate CO_2_ concentration. The beer was poured through two filter papers (Macherey-Nagel, 500713032 MN 713 ¼) to remove carbon dioxide and prevent spontaneous foaming. Samples were then prepared for measurements by targeted Headspace-Gas Chromatography-Flame Ionization Detector/Flame Photometric Detector (HS-GC-FID/FPD), Headspace-Solid Phase Microextraction-Gas Chromatography-Mass Spectrometry (HS-SPME-GC-MS), colorimetric analysis, enzymatic analysis, Near-Infrared (NIR) analysis, as described in the sections below. The mean values of biological duplicates are reported for each compound.

Specifically, HS-GC-FID/FPD (Shimadzu GC 2010 Plus) was used to measure higher alcohols, acetaldehyde, esters, 4-vinyl guaicol, and sulfur compounds. HS-SPME-GC-MS (Shimadzu GCMS-QP-2010 Ultra) was used to measure additional volatile compounds, mainly comprising terpenoids and esters. Discrete photometric and enzymatic analysis (Thermo Scientific Gallery Plus Beermaster Discrete Analyzer) was used to measure acetic acid, ammonia, beta-glucan, iso-alpha acids, color, sugars, glycerol, iron, pH, protein, and sulfite. NIR analysis (Anton Paar Alcolyzer Beer ME System) was used to measure ethanol.

#### Determining beer preference

A trap-based assay, adapted from,^62^ was used to determine insect preference to each beer. Traps were constructed from polysterene vials and polypropylene lids. Vials measured 4 cm x ø 3.5 cm, removable lids were 1.5 cm x ø 3.5 cm with a ø 1 cm hole in the center, and a funnel shaped entry opened into the trap at ø 0.5 cm (Figure 2A). This design prevented escape after flies had made their choice.

Prior to each assay, *D. suzukii* and *D. melanogaster* were starved in the dark, with water, for 6 h and 24 h respectively, beginning at less than 24 h post-emergence. The difference in starvation times is due to low response rates from *D. melanogaster* with only a 6-h starvation period and low survival rates of *D. suzukii* after 24 h of starvation (Figure S1). To measure survival, 100 flies of each species were placed in a starvation vial at an age less than 24 h old, with or without water. The number of flies still alive were counted at several time points to establish median survival time. As the majority of *D. melanogaster* survived past 72 h, no median survival time could be calculated. Experiment time (18 h) plus starvation time for *D. suzukii* and *D. melanogaster* were 24 h and 42 h, respectively, corresponding to an approximate survival rate of 95% at these time points.

Traps were filled with 5 mL of “blank” beer (Piedboeuf) or experimental beer. Lids were secured using Parafilm. One blank and one experimental trap were placed into two adjacent corners of a 15 × 15 × 15 cm arena and 100 flies were released from a ø 5 cm disc on the opposite side of the arena (Figure 2B). To minimize other visual cues, arenas were blocked on all sides except the top above which a single diffused overhead light was kept on for the duration of the experiment. After 18 h, the traps were removed and the number of flies in each trap were counted. Each beer was tested four times with differing trap arrangements to remove position bias.

#### Chemical synthesis of tobacarol

All chemicals for synthesis of tobacarol (dihydroisocaryophyllene epoxide) were purchased from Acros Organics and TCI Europe. For column chromatography, 70–230 mesh silica 60 (Acros) was used as the stationary phase. NMR spectra were recorded on a Bruker Avance III HD 400 spectrometer and chemical shifts (δ) were reported in parts per million (ppm) referenced to tetramethylsilane (^1^H), or the internal solvent signal (^13^C). A high-resolution mass spectrum was acquired on a quadrupole orthogonal acceleration time-of-flight mass spectrometer (Synapt G2 HDMS, Waters, Milford, MA). The sample was infused at 3*μ*L/min and the spectrum was obtained in positive ionization mode with a resolution of 15000 (FWHM) using leucine enkephalin as lock mass.

Dihydroisocaryophyllene epoxide was prepared in three steps from β-caryophyllene (Figure S4). β-Caryophyllene was isomerized using a modified literature procedure.^66^ In a round-bottom flask equipped with a reflux condenser, a mixture of β-caryophyllene (22.75 mmol, 4.65 g), ceric ammonium nitrate (2.05 mmol, 1.124 g) and acetonitrile (200 mL) was stirred at 80°C for 4 h under nitrogen atmosphere. Isocaryophyllene was extracted from the reaction mixture with pentane (400 mL and 250 mL). The combined pentane layers were washed with brine, dried over MgSO_4_ and concentrated to afford the isomerized product in 93% yield (4.31 g). According to a procedure given in the literature,^67^ isocaryophyllene oxide was synthesized in 64% yield (2.06 g, 2 diastereomers) from isocaryophyllene (14.68 mmol, 3 g) and meta-chloroperoxybenzoic acid (19.08 mmol, 4.39 g). Isocaryophyllene oxide was converted into the final product under mild hydrogenation conditions. To a round-bottom flask, the diastereomeric mixture of isocaryophyllene oxide (540 mg, 2.45 mmol), anhydrous tetrahydrofuran (6 mL) and Adams' catalyst PtO_2_ (17 mg, 75 μmol) were added. The flask was placed under H_2_ atmosphere (balloon pressure) and stirred at room temperature. After 70 h, the reaction mixture was filtered through a path of Celite, concentrated and purified by column chromatography (pentane/MTBE gradient, 0 to 3% MTBE (v/v)) on silica gel to afford dihydroisocaryophyllene oxide in 82% yield (449 mg, 4 diastereomers) as a colorless oil. HRMS (ESI-Q-TOF): m/z [M + H]+ calcd for C_15_H_26_O: 223.2056; found: 223.2056.

#### Attractiveness to key beer-related compounds

The same starvation scheme and traps were utilized for a compound screen assay adapted from.^43^ In this experiment, 12 traps were arranged in a 30 cm diameter circle inside a 45 × 45 × 45 cm arena (Figure 4A). Compounds were purchased from Sigma at the highest purity available, with two exceptions. Isomerized hop extract was purchased from Brouwland bvba (Beverlo, Belgium) and tobacarol (dihydroisocaryophyllene epoxide) was synthesized at the KU Leuven Department of Chemistry (see synthesis methods below). Each compound was spiked into blank beer at the highest concentration detected in the beer dataset. Concentrations can be found in Table S2. Within each arena, blank beer and compounds were present twice in opposite positions on the circle. The entire set was repeated once with new positions for each compound. For each replicate, approximately 800 flies were released into each arena. After 18 h, the number of flies caught per compound, per arena were counted and normalized to the number of flies caught in the blank beer trap within the same arena. Next, these values were averaged across the two arena’s; so that values below 100 indicate possible repellent effects, and values above 100 indicate possible attractive effects. Pilot assays demonstrated efficacy of the screen with a positive response (attraction) for high preference indexed beers (Palm and Cornet) and the known attractant β-cyclocitral (Figure S5A). No major position bias was seen after all compounds had been screened (Figure S5B).

#### Competition assays with synthetic lures

For testing of the synthetic lure, competition assays were performed between the two fly species. Drosotraps and Dros’Attract were purchased from Biobest Group NV (Westerlo, Belgium). Apple cider vinegar (L’Etoile) was purchased from a local grocery store. Per manufacturer’s instructions, 200mL of apple cider vinegar, Dros’Attract, or the synthetic lure was added to a Drosotrap and hung in the 45 × 45 × 45 cm arena. Flies were subjected to a 6-h starvation scheme and 200 individuals from each species (approximately 1:1 male:female) were simultaneously released into the arena. After 18 h, flies caught in the trap were sorted by sex and species. For lures using water or acetic acid + ethanol as a background, 0.01% Triton X-100 was added as a drowning solution.

#### Compound attractiveness in greenhouse

To test candidate compounds’ effects on *D. suzukii*’s attraction when applied to commercial lures, 250 × 150 × 200 cm insect cages were placed in a greenhouse compartment surrounded by tomato plants (*Solanum lycoperscium*) (Figures 7A and 7B). The average temperature in the greenhouse compartment during the experiment was 22 ± 2°C. Each arena contained four Drosotraps, with two controls containing Dros’Attract and two treatments containing Dros’Attract spiked with a single compound at concentrations found in Table S2. The volume of Dros’Attract used in both the treatment and control was 100 mL. The two controls and two treatment traps were placed diagonally opposite each other at 1-m height from the ground. No plants were present in the test arena. 25 individuals of each sex were released at ground level in the center of each arena at the age of 7–10 days. After 24 h, the flies were collected, counted and sexed for each trap. The experiment was repeated 15 times per compound over 30 experimental days, with differing trap arrangements to eliminate position bias.

#### Calcium imaging

Young female flies (3–8 days old) expressing *Orco>GCaMP6f* for optical imaging were prepared following the protocol previously described by.^50^ Initially, flies were immobilized by anesthetizing them on ice and securing them to a custom Plexiglas stage with a copper plate (Athene Grids, Plano). The proboscis was stabilized using a needle and the head was affixed to the stage using Protemp II adhesive (3M ESPE). Additionally, the antennae were gently extended forward using a fine metal wire. Subsequently, a plastic coverslip with a circular aperture was carefully positioned on the fly’s head. A two-component silicon material (World Precision Instruments) was applied around the circular window to prevent the Ringer’s solution from leaking onto the antennae. A small aperture beneath the Ringer’s solution, between the eyes and the ocelli of the fly’s head, was created to apply the Ringer’s solution (composed of NaCl: 130 mM, KCl: 5 mM, MgCl2: 2 mM, CaCl2: 2 mM, Sucrose: 36 mM, HEPES-NaOH (pH 7.3): 5 mM) during the experiment. Finally, visibility of the antennal lobe was enhanced, and light scattering minimized, by clearing the fat, trachea, and air sacs.

Calcium imaging experiments were conducted using a wide-field TillPhotonics imaging setup (TILL imago, http://www.till-photonics.com), which was equipped with a CCD camera (PCO imaging, http://www.pco.de) mounted on a fluorescence microscope (BX51WI, http://www.olympus.com). For calcium imaging, a 20-x water immersion objective (NA 0.95, XLUM Plan FI, http://www.olympus.com) was used. The samples were excited using a wavelength of 475 nm, following the methodology described by.^49^ Careful selection of a specific focal plane allowed to identify several Orco+ glomeruli located in the upper layer of the antennal lobe.

#### Odor application

10 μL of odorants (Sigma-Aldrich), diluted 1:10 in mineral oil (VWR Life Science), were pipetted onto a filter paper positioned inside glass Pasteur pipettes. For the imaging experiments a concentration of 10^−1^ (i.e., pure odor dilution 1:10 in solvent) was required in order to induce clear calcium responses, which was higher than the concentration applied in the behavioral assays. A stimulus controller (Stimulus Controller CS-55; Syntech) facilitated the application of odors. We employed a custom-made metal stage with a stainless steel tube to direct the airflow toward the fly’s antennae. This setup generated a continuous airflow of 1 L/min, accompanied by pulses of odor at a rate of 0.5 L/min. Odor stimuli were introduced into the airstream after a 2-s delay, and the application lasted for 2 s. During the imaging process, recordings were obtained at a frequency of 4 Hz, yielding a total of 40 frames (equivalent to a duration of 10 s).

In addition to the seven odors examined in this study, benzaldehyde was used as a diagnostic odor that possessed defined activation patterns, facilitating the identification of specific glomeruli. To ensure unbiased results and prevent habituation or interference in the odor responses, odors were applied in a randomized order, with a minimum interval of 1 min between successive stimulations.

### Quantification and statistical analyses

Statistical analyses results can be found in figure legends, results section and Tables S3–S8.

#### Beer compound analysis

Compound elution profiles were estimated using weighted nonnegative least squares, whereby library spectra of compounds identified on the basis of spectral match quality and retention index similarity were used as covariates.^68^ Batch effect correction was performed by normalizing against the internal standard compound 4-fluorobenzaldehyde. All data processing was carried out using the R programming languages. More details can be found in.^38^

#### Determining beer preference

Replicates with less than 50% response rates were discarded and repeated. Preference indices were calculated for each experimental beer by subtracting the number of flies in the blank beer from the number of flies in the experimental beer divided by the total number of flies caught. To test for significant differences, a Welch’s t-test was used.

#### Analysis of aroma data and fly preferences

Measurements of chemical properties (selected from^37^) and fly preference indices were scaled to zero mean and unit variance. Missing values for ammonia were imputed with the median measurement of the compound from all beers. Partial least-squares regression with 10-fold cross validation was performed with the kernel method. Data were analyzed and plotted using R (version 4.2.3), using R package *pls*^69^ (version 2.8–2) to analyze each fly species, with chemical properties as predictors and preference indices as targets.

#### Attraction to key beer-related compounds

The number of flies caught per compound, per arena were counted and normalized to the number of flies caught in the blank beer trap within the same arena. Next, these values were averaged across the two arenas; so that values below 100 indicate possible repellent effects, and values above 100 indicate possible attractive effects. These values, together with chemical diversity, were the two main criteria to select specific compounds for subsequent experiments.

#### Testing species-specific synthetic lures

Significance was calculated using one-way ANOVA with multiple comparisons between the background matrices and controls, as well as between lures with their respective backgrounds.

#### Compound attractiveness in greenhouse

Replicates with less than 30% response rate were discarded. Data were analyzed and plotted using R (version 4.2.3), using package tidyverse (version 2.0.0). Tests for significant differences between control and treatment groups were performed using the Wilcoxon signed-rank test, with the Holm-Bonferroni method for multiple corrections.

#### Calcium imaging analysis

Calcium imaging data were analyzed using FIJI (ImageJ 1.53a National Institutes of Health, USA). The StackReg Plugin was applied to correct the movement and converted the images to an 8-bit format for analysis. We manually outlined and established regions of interest (ROIs) for all visible glomeruli, identifying them by comparing with the AL atlas specific to each species. Additionally, the identification of glomeruli was aided by examining their responses to diagnostic odors. The mean fluorescence for each ROI across all 40 frames was measured. To calculate ΔF/F, the averaged values of frames 0 to 8 (representing the pre-odor stimulation fluorescence background) were subtracted and then each ΔF was normalized to the raw fluorescence signal. The peak ΔF/F was determined as the mean of the 6 frames with the highest response during the odor application period, which could vary between species and samples.

To generate the false color-coded images (Figure 5) in FIJI, we subtracted the basal background (average Z projection of frames 1 to 8) and then calculated the mean response of frames 10 to 16. Further, the data were normalized with the raw fluorescent signal. Each set of images corresponds to an experiment conducted on a single fly. We then applied a 16-color scale to each image. We corrected the data for further analysis to enable valid comparisons between species. Specifically, the overall signal intensities exhibit some variations between species, likely partially attributed to differences in the expression of the GCaMP6f protein. In order to correct for expression differences, the odor-evoked responses in each fly were normalized to its own maximum response defined as normalized ΔF/F in Figure 5. Using the mean responses for each odor/glomeruli combination between the two species, heat maps (normalized ΔF/F) were generated in GraphPad.

We conducted statistical analyses using the GraphPad Prism 9.0.2 software (GraphPad), considering *p* < 0.05 as the threshold for statistical significance. The specific statistical tests and any special conditions for each experiment are provided in the figure legends. For figure preparation, we utilized a combination of FIJI, Graphpad, Microsoft Excel 2016, and Adobe Photoshop.
