# Supplementary material for: Drosophila suzukii and Drosophila melanogaster prefer distinct microbial and plant aroma compounds in a complex fermented matrix
Source: iScience. 2024 Oct 10;27(11):111141. doi: 10.1016/j.isci.2024.111141 (PMC11549995; doi:10.1016/j.isci.2024.111141)
Supplement: Document S1. Figures S1–S6 and Tables S1–S8 [file mmc1.pdf]

iScience, Volume *27*

Supplemental information

Drosophila suzukii and *Drosophila melanogaster* prefer distinct microbial and plant aroma compounds in a complex fermented matrix

Maria C. Dzialo, Somasundar Arumugam, Supinya Piampongsant, Lloyd Cool, Christophe Vanderaa, Beatriz Herrera-Malaver, Tomas Opsomer, Wim Dehaen, Tom Wenseleers, Miguel Roncoroni, Amani Alawamleh, Felix Wäckers, Bart Lievens, Bill S. Hansson, Karin Voordeckers, Silke Sachse, and Kevin J. Verstrepen

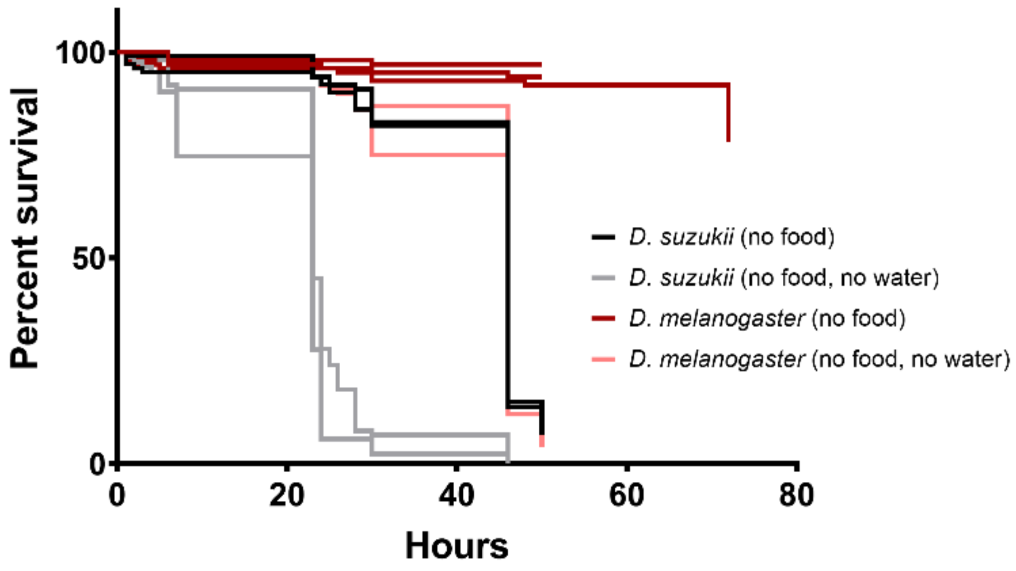

Figure S1. *D. melanogaster* survive starvation longer than *D. suzukii*. Related to Figure 2. Median survival: *D. suzukii* (no food) = 46 hr, *D. suzukii* (no food, no water) = 23 hr, *D. melanogaster* (no food) >72 hr, *D. melanogaster* (no food, no water) = 46 hr.

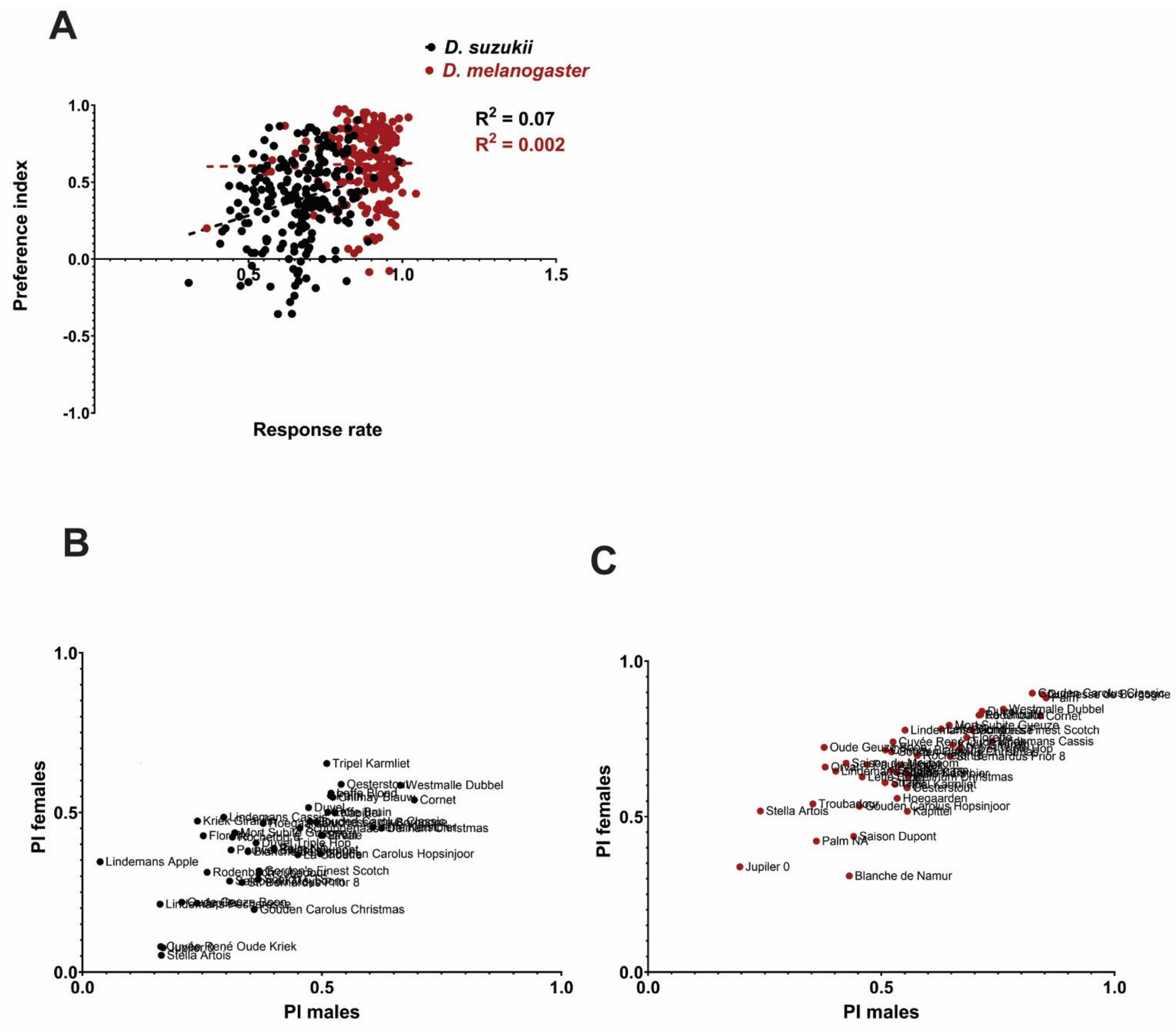

Figure S2. Additional data on preference indices for *D. melanogaster* and *D. suzukii* for the different beers tested. Related to Figure 2. (A) Response rates and corresponding preference indices for all replicates performed for assays shown in **Figure 2**. R-squared values are shown for each species and represent goodness of fit of a linear regression. (*D. suzukii* black and *D. melanogaster* red) and overall (grey). Preference indices broken down by sex for (B) *D. suzukii* and (C) *D. melanogaster*.

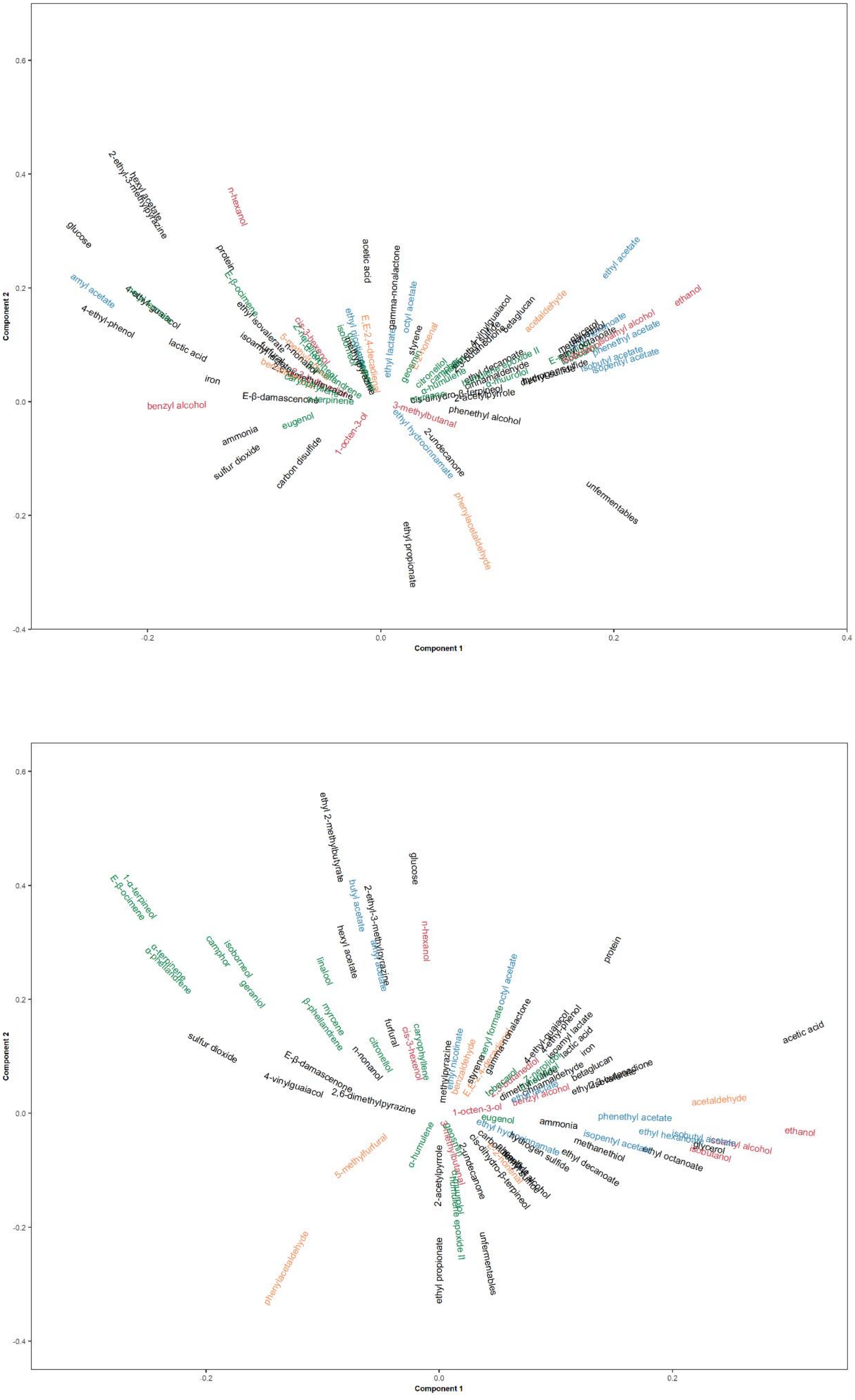

Figure S3. Full set of PLS loadings plots with all compounds for *D. suzukii* (top panel) and *D. melanogaster* (bottom panel). Related to Figure 3. Labels are colored according to compound type (green = terpenoid, blue = esters, red = alcohols, orange = aldehydes, black = misc.).

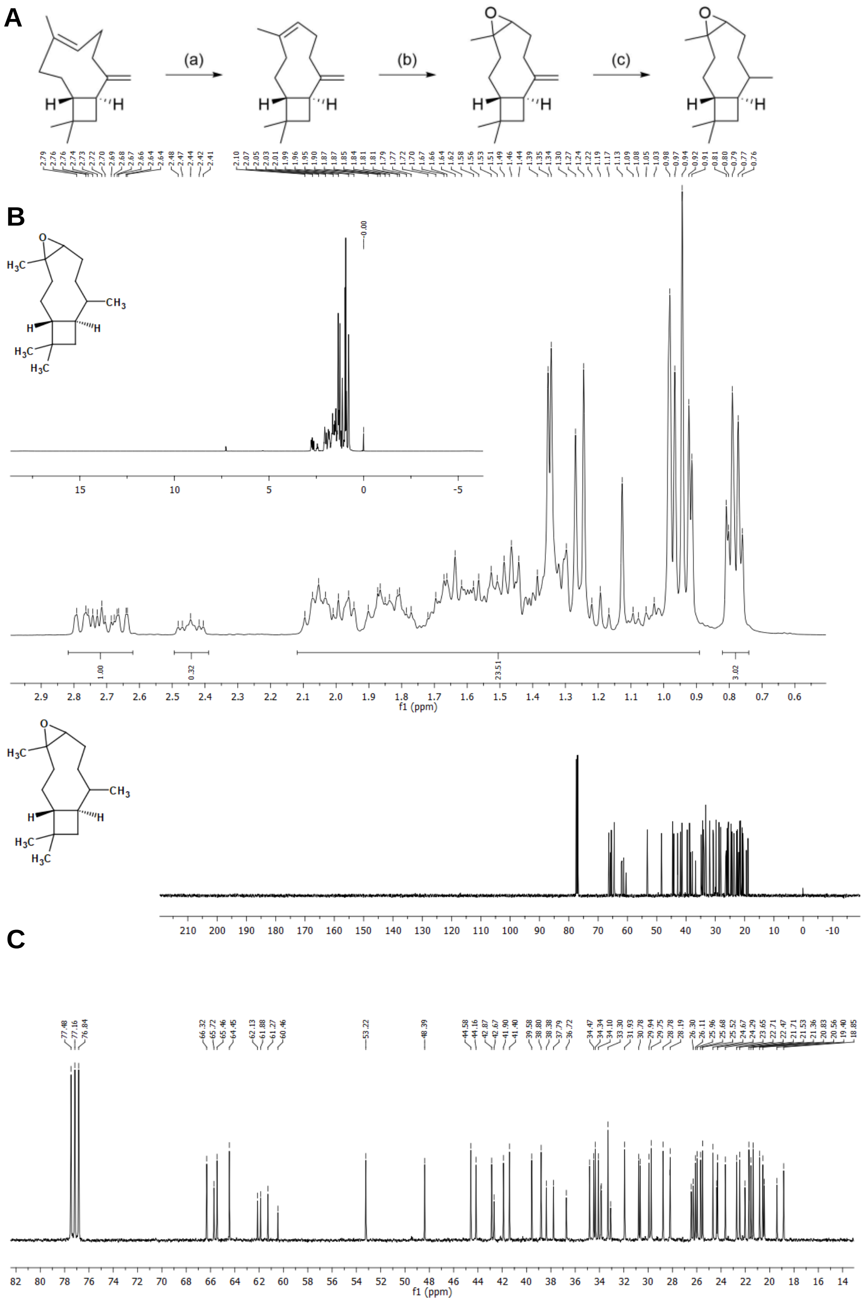

Figure S4: Synthesis and NMR spectra of dihydroisocaryophyllene oxide (tobacarol). Related to Figure 4.
(A) Synthetic pathway; reagents and conditions: (a) CAN, ACN, 80 °C, 4 h. (b) *m*CPBA, DCM, rt, 4 h. (c) PtO_2, H_2, THF, rt, 70 h. (B) ^{1}H NMR spectrum of dihydroisocaryophyllene oxide (4 diastereomers) ($CDCl_3$, 400 MHz): δ 2.82 – 2.62 (m), 2.50 – 2.39 (m), 2.13 – 0.88 (m), 0.83 – 0.73 (m). (C) ^{13}C NMR spectrum of dihydroisocaryophyllene oxide (4 diastereomers) ($CDCl_3$, 101 MHz): δ 66.3, 65.7, 65.5, 64.5, 62.1, 61.9, 61.3, 60.5, 53.2, 48.4, 44.6, 44.2, 42.9, 42.7, 41.9, 41.4, 39.6, 38.8, 38.4, 37.8, 36.7, 34.8, 34.5, 34.3, 34.1, 33.9, 33.9, 33.3, 33.1, 31.9, 30.8, 30.7, 29.9, 29.8, 28.8, 28.2, 28.2, 26.5, 26.3, 26.1, 26.0, 25.7, 25.5, 24.7, 24.4, 24.3, 23.7, 22.7, 22.5, 22.0, 21.7, 21.5, 21.4, 20.8, 20.6, 20.5, 19.4, 18.9.

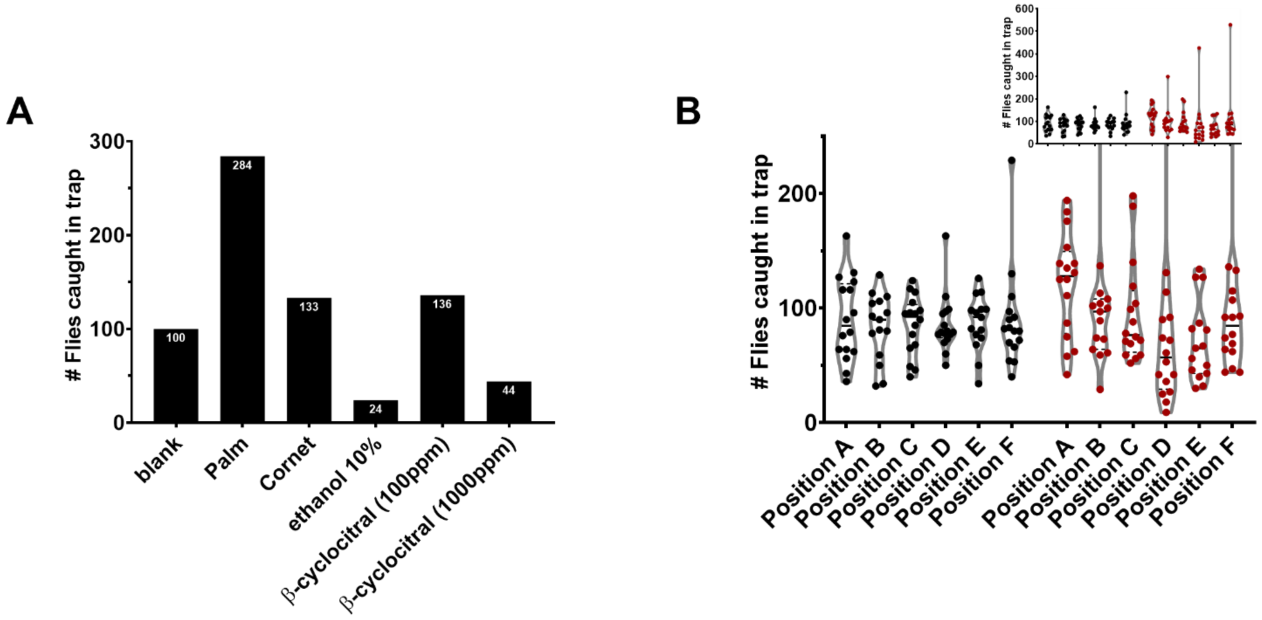

Figure S5. Pilot arena assays demonstrate efficacy of compound screen as well as lack of position bias. Related to Figure 4. A) Pilot assay of arena assay using *D. suzukii* against known or potential attractants. Blank is unspiked control beer (Piedboeuf Blond). (B) Position bias of arena assay for *D. suzukii* (black) and *D. melanogaster* (red). Inset shows the outliers of *D. melanogaster* acetaldehyde tests. One-way ANOVA with multiple comparison tests showed no significant difference between positions (**Table S3**).

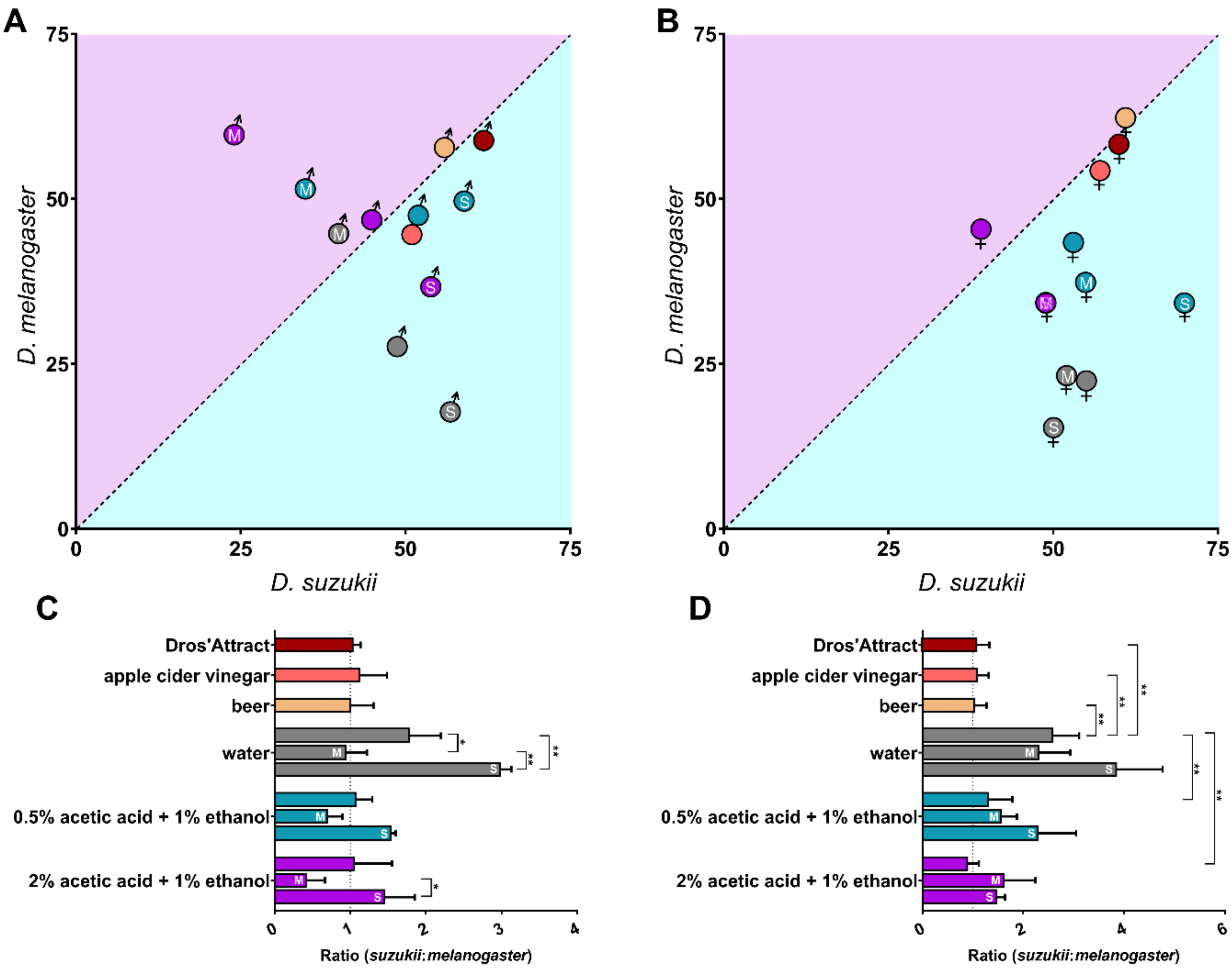

Figure S6. Number of flies caught in Droso'Trap assays of Figure 6 broken down by sex. Related to Figure 6. A. Total number of *D. suzukii* males vs *D. melanogaster* males caught in the same trap. **B**. Total number of *D. suzukii* females vs *D. melanogaster* females caught in the same trap. Colors of data points correspond to the background matrix as indicated in panel C and D. Data points with "S" contain linalool, geraniol, dimethyl sulfide, and β-cyclocitral. Data points with "M" contain pentyl acetate, ethyl acetate, isoamyl acetate, and isoamyl alcohol. (**C, D**) Ratio of *D. suzukii* to *D. melanogaster* caught in the same trap (mean ± SD, n = 3). Bar colors correspond to those in panel A. Significance was calculated using one-way ANOVA with multiple comparisons between the background matrices and controls as well as between lures with their respective backgrounds (**Table S4-S8**). *$p < 0.05$, **$p < 0.01$

Table S1. List of beers tested for drosophilid preference. Related to Figure 1 and Figure 2.

Category	Beer
**Amber**	Palm
	Pauwel Kwak
**Blond**	Kapittel Blond
	Leffe Blond
	La Chouffe
**Brett**	Orval
	Schuppenaas
**Brown**	Gouden Carolus Classic
	Leffe Bruin
	St. Bernardus Prior 8
**Christmas**	Delirium Christmas
	Gouden Carolus Christmas
	Leffe Kerst
**Dubbel**	Floreffe Dubbel
	Westmalle Dubbel
**Flemish Ales**	Duchesse de Bourgogne
	Rodenbach
**Fruit Beer**	Lindemans Apple
	Lindemans Cassis
**Geuze**	Mort Subite Gueuze
	Oude Geuze Boon
**Hoppy**	Duvel Triple Hop
	Gouden Carolus Hopsinjoor
**Kriek**	Cuvée Rene Oude Kriek
	Kriek Girardin
	Lindemans Pêcheresse
**Low-No Alcohol**	Jupiler 0.0
	Palm NA
	Piedbœuf Blond
**Pils**	Jupiler
	Stella Artois
**Saison**	Saison du Meyboom
	Saison Dupont
**Scotch**	Gordon’s Finest Scotch
	Scotch CTS
**Stout**	Oesterstout
	Troubadour Obscura
**Strong Ale**	Chimay Blauw
	Rochefort 8
**Tripel**	Cornet
	Duvel
	Straffe Hendrik
	Tripel Karmeliet
**Witbier**	Blanche de Namur
	Hoegaarden

Table S2. Compounds tested in the arena trap assay. Related to Figure 4.

Compound	Method of detection (in beer)[a]	Compound Category[b]	Concentration[c]	Odor[d]	Natural Occurrence[e]	Main Source in beer[f]
1-octen-3-ol	GC-MS	alcohol	0.07 ppm	earthy	mushroom, clover, wheat, herbs, spices	Wheat, spices
2,3-butanediol	GC-FID	alcohol	0.50 ppm	creamy, buttery	cocoa butter	yeast
2,3-butanedione (diacetyl)	GC-MS	ketone	0.50 ppm	buttery	butter, vinegar, coffee	Yeast
4-ethyl-guaiacol	GC-MS	phenol	3.6 ppm	spicy, clove-like, medicinal	arabica coffee, plants	Yeast
4-ethyl-phenol	GC-MS	phenol	1.0 ppm	smoky, phenolic	arabica coffee	Yeast
4-vinylguaiacol	GC-MS	phenol	0.68 ppm	woody, clove	buckwheat	Yeast
acetaldehyde	GC-FID	aldehyde	19 ppm	fresh, fruity, musty	plants, tobacco smoke, microbes	Yeast
acetic acid	Enzymatic assay	acid	33 g/L	acidic, vinegar		Yeast, bacteria
α-humulene	GC-MS	terpenoid	0.019 ppm	woody	pine, orange, tobacco	Hops
ammonia	Enzymatic assay	inorganic	200 mg/L	Urine, sweat	decomposing organic material	Barley (malt), hops, yeast
benzaldehyde	GC-MS	aldehyde	1.9 ppm	fruity, almond, cherry	Almonds, stonefruits	Yeast
β-caryophyllene	GC-MS	terpenoid	0.034 ppm	woody, spice, clove	rosemary, cannabis	Hops
β-cyclocitral	GC-MS	terpenoid	100 ppm	tropical, saffron, herbal	saffron	Hops
camphor	GC-MS	terpenoid	1.4 ppm	woody	camphor laurel wood, rosemary	Hops
diethyl sulfide	GC-FPD	sulfur	4.7 ppb	sulfurous	durian, potatoes	Barley
dimethyl sulfide	GC-FPD	sulfur	154 ppb	sulfury, onion, cabbage	cooked vegetables	Barley, yeast, bacteria
ethanol	Spectrophotometric	alcohol	11.6%	alcoholic, medical		yeast
ethyl acetate	GC-FID	ester	130 ppm	fruity, sweet, nail polish	fruit	yeast
ethyl isovalerate	GC-FID	ester	0.14 ppm	fruity, pineapple	Fruits, vegetables	yeast

ethyl lactate	GC-MS	ester	0.12 ppm	tart, fruity, butterscotch	Various fruits	yeast
eugenol	GC-MS	terpenoid	0.89 ppm	spicy, clove, woody	clove, nutmeg, cinnamon, bay leaf	hops
γ-nonalactone	GC-MS	ketone	0.22 ppm	coconut	bourbon whiskey	yeast
geosmin	GC-MS	terpenoid	0.0040 ppm	earthy	beets, petrichor	yeast
geraniol	GC-MS	terpenoid/alcohol	1.5 ppm	floral, fruity, rose, citrus	rose oil, citronella oil	hops
isoamyl alcohol	GC-FID	alcohol	102 ppm	alcoholic, banana	black truffles	yeast
isoamyl lactate	GC-MS	ester	0.72 ppm	fruity, creamy, nutty		Yeast
isopentyl acetate (isoamyl acetate)	GC-FID	ester	13.1 ppm	banana, fruity	honey bee stings	Yeast
Isomerized hop extract	NA	acid	6%	none		Hops
lactic acid	Enzymatic assay	acid	5.9 g/L	none	Sour milk products such as yoghurt	bacteria
linalool	GC-MS	terpenoid	6.6 ppm	citrus, floral, woody	mint, laurels, cinnamon, rosewood, citrus	hops
myrcene	GC-MS	terpenoid	0.11 ppm	peppery, spicy, balsam	plant oils	hops
nerolidol	GC-MS	terpenoid	0.077 ppm	floral, citrus, woody	bay, cannabis, thyme, parsley, cardamom	hops
pentyl acetate	GC-MS	ester	0.016 ppm	fruity, pear, apple	fruit	Yeast
phenylacetaldehyde	GC-MS	aldehyde	0.064 ppm	honey, floral, chocolate	chocolate, buckwheat, flowers	Yeast

a. Mode of detection of compound in beer from [S1,S2]
b. Category names used for color coding in **Figure 3** and **Figure S3**.
c. Concentrations correspond to levels detected in beer from [S1,S2].
d. Odor descriptors from The Good Scents database http://www.thegoodscentscompany.com
e. Occurrence of compounds; apart from beer fermentation.
f. Main source of compound in beer. Yeast= produced by yeast during fermentation.

Table S3. One-way ANOVA and multiple comparison test between different arena positions. Related to Figure 4.

	D. suzukii					
	A	B	C	D	E	F
A		0.99268886	0.99789999	0.99875088	0.99972513	0.99999956
B			0.99999227	0.99996977	0.99976729	0.99629917
C				0.99999997	0.99999143	0.99919017
D					0.99999864	0.99957727
E						0.99994114
F						
	D. melanogaster					
	A	B	C	D	E	F
A		0.97137487	0.92246393	0.66383803	0.41550297	0.99893184
B			0.99996151	0.98091684	0.88057023	0.99865772
C				0.99525598	0.93952189	0.99065007
D					0.99843477	0.87110261
E						0.65547625
F						

Table S4. One-way ANOVA and multiple comparison test between species ratios for different background matrices. Related to Figure 6.

	Dros'Attract	apple cider vinegar	beer	water	0.5% acetic acid + 1% ethanol	2% acetic acid + 1% ethanol
**Dros'Attract**		0.99979	0.99706	0.00011	0.98540	0.97347
**apple cider vinegar**			0.97692	0.00015	0.99870	0.91089
**beer**				0.00006	0.87634	0.99968
**water**					0.00026	0.00004
**0.5% acetic acid + 1% ethanol**						0.74077
**2% acetic acid + 1% ethanol**						

Significant *p* values highlighted in yellow

Table S5. One-way ANOVA and multiple comparison test between species ratios for different lures and respective background matrices. Related to Figure 6.

	water	water + S	water + M
**water**		0.00115	0.01961
**water + S**			0.00010
**water + M**			
	**0.5% acetic acid + 1% ethanol**	**0.5% acetic acid + 1% ethanol + S**	**0.5% acetic acid + 1% ethanol + M**
**0.5% acetic acid + 1% ethanol**		0.06678	0.73048
**0.5% acetic acid + 1% ethanol + S**			0.02608
**0.5% acetic acid + 1% ethanol + M**			
	**2% acetic acid + 1% ethanol**	**2% acetic acid + 1% ethanol + S**	**2% acetic acid + 1% ethanol + M**
**2% acetic acid + 1% ethanol**		0.04475	0.52709
**2% acetic acid + 1% ethanol + S**			0.01208
**2% acetic acid + 1% ethanol + M**			

Significant *p* values highlighted in yellow

Table S6. One-way ANOVA and multiple comparison test between total number of *D. suzukii* caught for different lures and respective background matrices. Related to Figure 6.

**Tukey's multiple comparisons test**	Mean Diff,	95,00% CI of diff,	Below threshold?	Summary	Adjusted P Value
apple cider vinegar vs. Dros'Attract	-13	-60,42 to 34,42	No	ns	0,9987
apple cider vinegar vs. water	4,333	-43,08 to 51,75	No	ns	>0,9999
apple cider vinegar vs. beer	-8,667	-56,08 to 38,75	No	ns	>0,9999
apple cider vinegar vs. matrix 1	3	-44,42 to 50,42	No	ns	>0,9999
apple cider vinegar vs. matrix 2	24,67	-22,75 to 72,08	No	ns	0,8079
apple cider vinegar vs. Lure-S (water)	1,667	-45,75 to 49,08	No	ns	>0,9999
apple cider vinegar vs. Lure-S (beer)	-6	-53,42 to 41,42	No	ns	>0,9999
apple cider vinegar vs. Lure-S (matrix 1)	-20	-67,42 to 27,42	No	ns	0,9465
apple cider vinegar vs. Lure-S (matrix 2)	5	-42,42 to 52,42	No	ns	>0,9999
apple cider vinegar vs. Lure-M (water)	15,67	-31,75 to 63,08	No	ns	0,9923
apple cider vinegar vs. Lure-M (beer)	4,667	-42,75 to 52,08	No	ns	>0,9999
apple cider vinegar vs. Lure-M (matrix 1)	19	-28,42 to 66,42	No	ns	0,9631
apple cider vinegar vs. Lure-M (matrix 2)	35,67	-11,75 to 83,08	No	ns	0,302
Dros'Attract vs. water	17,33	-30,08 to 64,75	No	ns	0,9819
Dros'Attract vs. beer	4,333	-43,08 to 51,75	No	ns	>0,9999
Dros'Attract vs. matrix 1	16	-31,42 to 63,42	No	ns	0,9908
Dros'Attract vs. matrix 2	37,67	-9,751 to 85,08	No	ns	0,2322
Dros'Attract vs. Lure-S (water)	14,67	-32,75 to 62,08	No	ns	0,9958
Dros'Attract vs. Lure-S (beer)	7	-40,42 to 54,42	No	ns	>0,9999
Dros'Attract vs. Lure-S (matrix 1)	-7	-54,42 to 40,42	No	ns	>0,9999
Dros'Attract vs. Lure-S (matrix 2)	18	-29,42 to 65,42	No	ns	0,9756
Dros'Attract vs. Lure-M (water)	28,67	-18,75 to 76,08	No	ns	0,6239
Dros'Attract vs. Lure-M (beer)	17,67	-29,75 to 65,08	No	ns	0,9789
Dros'Attract vs. Lure-M (matrix 1)	32	-15,42 to 79,42	No	ns	0,46
Dros'Attract vs. Lure-M (matrix 2)	48,67	1,249 to 96,08	Yes	*	0,0401
water vs. beer	-13	-60,42 to 34,42	No	ns	0,9987
water vs. matrix 1	-1,333	-48,75 to 46,08	No	ns	>0,9999
water vs. matrix 2	20,33	-27,08 to 67,75	No	ns	0,94
water vs. Lure-S (water)	-2,667	-50,08 to 44,75	No	ns	>0,9999
water vs. Lure-S (beer)	-10,33	-57,75 to 37,08	No	ns	0,9999
water vs. Lure-S (matrix 1)	-24,33	-71,75 to 23,08	No	ns	0,8211
water vs. Lure-S (matrix 2)	0,6667	-46,75 to 48,08	No	ns	>0,9999
water vs. Lure-M (water)	11,33	-36,08 to 58,75	No	ns	0,9997
water vs. Lure-M (beer)	0,3333	-47,08 to 47,75	No	ns	>0,9999
water vs. Lure-M (matrix 1)	14,67	-32,75 to 62,08	No	ns	0,9958
water vs. Lure-M (matrix 2)	31,33	-16,08 to 78,75	No	ns	0,4919
beer vs. matrix 1	11,67	-35,75 to 59,08	No	ns	0,9996
beer vs. matrix 2	33,33	-14,08 to 80,75	No	ns	0,3986
beer vs. Lure-S (water)	10,33	-37,08 to 57,75	No	ns	0,9999

beer vs. Lure-S (beer)	2,667	-44,75 to 50,08	No	ns	>0,9999
beer vs. Lure-S (matrix 1)	-11,33	-58,75 to 36,08	No	ns	0,9997
beer vs. Lure-S (matrix 2)	13,67	-33,75 to 61,08	No	ns	0,9978
beer vs. Lure-M (water)	24,33	-23,08 to 71,75	No	ns	0,8211
beer vs. Lure-M (beer)	13,33	-34,08 to 60,75	No	ns	0,9983
beer vs. Lure-M (matrix 1)	27,67	-19,75 to 75,08	No	ns	0,673
beer vs. Lure-M (matrix 2)	44,33	-3,084 to 91,75	No	ns	0,0844
matrix 1 vs. matrix 2	21,67	-25,75 to 69,08	No	ns	0,9088
matrix 1 vs. Lure-S (water)	-1,333	-48,75 to 46,08	No	ns	>0,9999
matrix 1 vs. Lure-S (beer)	-9	-56,42 to 38,42	No	ns	>0,9999
matrix 1 vs. Lure-S (matrix 1)	-23	-70,42 to 24,42	No	ns	0,8691
matrix 1 vs. Lure-S (matrix 2)	2	-45,42 to 49,42	No	ns	>0,9999
matrix 1 vs. Lure-M (water)	12,67	-34,75 to 60,08	No	ns	0,999
matrix 1 vs. Lure-M (beer)	1,667	-45,75 to 49,08	No	ns	>0,9999
matrix 1 vs. Lure-M (matrix 1)	16	-31,42 to 63,42	No	ns	0,9908
matrix 1 vs. Lure-M (matrix 2)	32,67	-14,75 to 80,08	No	ns	0,4288
matrix 2 vs. Lure-S (water)	-23	-70,42 to 24,42	No	ns	0,8691
matrix 2 vs. Lure-S (beer)	-30,67	-78,08 to 16,75	No	ns	0,5245
matrix 2 vs. Lure-S (matrix 1)	-44,67	-92,08 to 2,751	No	ns	0,0799
matrix 2 vs. Lure-S (matrix 2)	-19,67	-67,08 to 27,75	No	ns	0,9525
matrix 2 vs. Lure-M (water)	-9	-56,42 to 38,42	No	ns	>0,9999
matrix 2 vs. Lure-M (beer)	-20	-67,42 to 27,42	No	ns	0,9465
matrix 2 vs. Lure-M (matrix 1)	-5,667	-53,08 to 41,75	No	ns	>0,9999
matrix 2 vs. Lure-M (matrix 2)	11	-36,42 to 58,42	No	ns	0,9998
Lure-S (water) vs. Lure-S (beer)	-7,667	-55,08 to 39,75	No	ns	>0,9999
Lure-S (water) vs. Lure-S (matrix 1)	-21,67	-69,08 to 25,75	No	ns	0,9088
Lure-S (water) vs. Lure-S (matrix 2)	3,333	-44,08 to 50,75	No	ns	>0,9999
Lure-S (water) vs. Lure-M (water)	14	-33,42 to 61,42	No	ns	0,9973
Lure-S (water) vs. Lure-M (beer)	3	-44,42 to 50,42	No	ns	>0,9999
Lure-S (water) vs. Lure-M (matrix 1)	17,33	-30,08 to 64,75	No	ns	0,9819
Lure-S (water) vs. Lure-M (matrix 2)	34	-13,42 to 81,42	No	ns	0,3694
Lure-S (beer) vs. Lure-S (matrix 1)	-14	-61,42 to 33,42	No	ns	0,9973
Lure-S (beer) vs. Lure-S (matrix 2)	11	-36,42 to 58,42	No	ns	0,9998
Lure-S (beer) vs. Lure-M (water)	21,67	-25,75 to 69,08	No	ns	0,9088
Lure-S (beer) vs. Lure-M (beer)	10,67	-36,75 to 58,08	No	ns	0,9998
Lure-S (beer) vs. Lure-M (matrix 1)	25	-22,42 to 72,42	No	ns	0,7943
Lure-S (beer) vs. Lure-M (matrix 2)	41,67	-5,751 to 89,08	No	ns	0,1294
Lure-S (matrix 1) vs. Lure-S (matrix 2)	25	-22,42 to 72,42	No	ns	0,7943
Lure-S (matrix 1) vs. Lure-M (water)	35,67	-11,75 to 83,08	No	ns	0,302
Lure-S (matrix 1) vs. Lure-M (beer)	24,67	-22,75 to 72,08	No	ns	0,8079
Lure-S (matrix 1) vs. Lure-M (matrix 1)	39	-8,417 to 86,42	No	ns	0,1927
Lure-S (matrix 1) vs. Lure-M (matrix 2)	55,67	8,249 to 103,1	Yes	*	0,011
Lure-S (matrix 2) vs. Lure-M (water)	10,67	-36,75 to 58,08	No	ns	0,9998
Lure-S (matrix 2) vs. Lure-M (beer)	-0,3333	-47,75 to 47,08	No	ns	>0,9999
Lure-S (matrix 2) vs. Lure-M (matrix 1)	14	-33,42 to 61,42	No	ns	0,9973

Lure-S (matrix 2) vs. Lure-M (matrix 2)	30,67	-16,75 to 78,08	No	ns	0,5245
Lure-M (water) vs. Lure-M (beer)	-11	-58,42 to 36,42	No	ns	0,9998
Lure-M (water) vs. Lure-M (matrix 1)	3,333	-44,08 to 50,75	No	ns	>0,9999
Lure-M (water) vs. Lure-M (matrix 2)	20	-27,42 to 67,42	No	ns	0,9465
Lure-M (beer) vs. Lure-M (matrix 1)	14,33	-33,08 to 61,75	No	ns	0,9966
Lure-M (beer) vs. Lure-M (matrix 2)	31	-16,42 to 78,42	No	ns	0,5082
Lure-M (matrix 1) vs. Lure-M (matrix 2)	16,67	-30,75 to 64,08	No	ns	0,9869

Table S7. One-way ANOVA and multiple comparison test between species ratios (males and females) for different background matrices. Related to Figure 6.

	MALES					
	**Dros'Attract**	**apple cider vinegar**	**beer**	**water**	**0.5% acetic acid + 1% ethanol**	**2% acetic acid + 1% ethanol**
**Dros'Attract**		0.99877	1.00000	0.13619	0.99999	1.00000
**apple cider vinegar**			0.99715	0.24006	0.99985	0.99977
**beer**				0.12142	0.99993	0.99996
**water**					0.16745	0.16134
**0.5% acetic acid + 1% ethanol**						1.00000
**2% acetic acid + 1% ethanol**						
	**FEMALES**					
	**Dros'Attract**	**apple cider vinegar**	**beer**	**water**	**0.5% acetic acid + 1% ethanol**	**2% acetic acid + 1% ethanol**
**Dros'Attract**		1.00000	0.99999	0.00246	0.96677	0.98791
**apple cider vinegar**			0.99997	0.00260	0.97257	0.98458
**beer**				0.00197	0.93551	0.99625
**water**					0.00873	0.00095
**0.5% acetic acid + 1% ethanol**						0.73328
**2% acetic acid + 1% ethanol**						

Significant *p* values highlighted in yellow

Table S8. One-way ANOVA and multiple comparison test between species ratios (males and females) for different lures and respective background matrices. Related to Figure 6.

	MALES				FEMALES		
	water	water + S	water + M		water	water + S	water + M
**water**		0.00671	0.03159			0.15740	0.90567
**water + S**			0.00040				0.09125
**water + M**							
	**0.5% acetic acid + 1% ethanol**	**0.5% acetic acid + 1% ethanol + S**	**0.5% acetic acid + 1% ethanol + M**		**0.5% acetic acid + 1% ethanol**	**0.5% acetic acid + 1% ethanol + S**	**0.5% acetic acid + 1% ethanol + M**
**0.5% acetic acid + 1% ethanol**		0.842837	0.322613			0.176691	0.844168
**0.5% acetic acid + 1% ethanol + S**			0.160020				0.352509
**0.5% acetic acid + 1% ethanol + M**							
	**2% acetic acid + 1% ethanol**	**2% acetic acid + 1% ethanol + S**	**2% acetic acid + 1% ethanol + M**		**2% acetic acid + 1% ethanol**	**2% acetic acid + 1% ethanol + S**	**2% acetic acid + 1% ethanol + M**
**2% acetic acid + 1% ethanol**		0.51360	0.19221			0.289587	0.159752
**2% acetic acid + 1% ethanol + S**			0.04424				0.884687
**2% acetic acid + 1% ethanol + M**							

Significant *p* values highlighted in yellow

SUPPLEMENTARY INFORMATION REFERENCES

S1. Roncoroni, M. & Verstrepen, K. *Belgian Beer: Tested and Tasted*. (Lannoo Publishers, 2018).

S2. Schreurs, M., Piampongsant, S., Roncoroni, M., Cool, L., Herrera-Malaver, B., Vanderaa, C., Theßeling, F. A., Kreft, Ł., Botzki, A., Malcorps, P., Daenen, L., Wenseleers, T. & Verstrepen, K. J. Predicting and improving complex beer flavor through machine learning. *Nat. Commun.* **15,** 2368 (2024).
